# Describing the Prevalence of Neural Tube Defects Worldwide: A Systematic Literature Review

**DOI:** 10.1371/journal.pone.0151586

**Published:** 2016-04-11

**Authors:** Ibrahim Zaganjor, Ahlia Sekkarie, Becky L. Tsang, Jennifer Williams, Hilda Razzaghi, Joseph Mulinare, Joseph E. Sniezek, Michael J. Cannon, Jorge Rosenthal

**Affiliations:** 1 National Center on Birth Defects and Developmental Disabilities, Centers for Disease Control and Prevention, Atlanta, Georgia, United States of America; 2 Carter Consulting Inc., Atlanta, Georgia, United States of America; Hospital de Especialidades del Niño y la Mujer de Queretaro, MEXICO

## Abstract

**Background:**

Folate-sensitive neural tube defects (NTDs) are an important, preventable cause of morbidity and mortality worldwide. There is a need to describe the current global burden of NTDs and identify gaps in available NTD data.

**Methods and Findings:**

We conducted a systematic review and searched multiple databases for NTD prevalence estimates and abstracted data from peer-reviewed literature, birth defects surveillance registries, and reports published between January 1990 and July 2014 that had greater than 5,000 births and were not solely based on mortality data. We classified countries according to World Health Organization (WHO) regions and World Bank income classifications. The initial search yielded 11,614 results; after systematic review we identified 160 full text manuscripts and reports that met the inclusion criteria. Data came from 75 countries. Coverage by WHO region varied in completeness (i.e., % of countries reporting) as follows: African (17%), Eastern Mediterranean (57%), European (49%), Americas (43%), South-East Asian (36%), and Western Pacific (33%). The reported NTD prevalence ranges and medians for each region were: African (5.2–75.4; 11.7 per 10,000 births), Eastern Mediterranean (2.1–124.1; 21.9 per 10,000 births), European (1.3–35.9; 9.0 per 10,000 births), Americas (3.3–27.9; 11.5 per 10,000 births), South-East Asian (1.9–66.2; 15.8 per 10,000 births), and Western Pacific (0.3–199.4; 6.9 per 10,000 births). The presence of a registry or surveillance system for NTDs increased with country income level: low income (0%), lower-middle income (25%), upper-middle income (70%), and high income (91%).

**Conclusions:**

Many WHO member states (120/194) did not have any data on NTD prevalence. Where data are collected, prevalence estimates vary widely. These findings highlight the need for greater NTD surveillance efforts, especially in lower-income countries. NTDs are an important public health problem that can be prevented with folic acid supplementation and fortification of staple foods.

## Introduction

Neural tube defects (NTDs), serious birth defects of the brain and spine, are a major, preventable public health burden. Globally, it is estimated that approximately 300,000 babies are born each year with NTDs [1], resulting in approximately 88,000 deaths and 8.6 million disability-adjusted life years (DALYs) [2, 3]. In low income countries, NTDs may account for 29% of neonatal deaths due to observable birth defects [4]. As morbidity and mortality from infectious diseases are decreasing worldwide, the contribution of birth defects to under-5 morbidity and mortality will continue to increase proportionally [5].

Conclusive evidence from clinical trials has led to recommendations for adequate periconceptional folic acid intake to reduce the occurrence of a NTD-affected pregnancy [6]; as a result, mandatory folic acid fortification (FAF) of staple cereal grains has been legislated in many countries as recently reviewed [7, 8]. Long-term surveillance of NTDs in countries that have successfully implemented fortification, such as the United States, Canada, Costa Rica, South Africa, and Chile, and data from a supplementation program in China suggest that folic acid interventions can reduce NTD prevalence to as low as 5–6 per 10,000 pregnancies [8, 9].

Because birth defects are a major cause of under-5 mortality, adequate surveillance data are needed for prevention and evaluation purposes. This is particularly important for birth defects that have well-established interventions. For example, depending on the baseline prevalence, it is estimated that the majority of NTDs can be prevented with folic acid [4, 10]. However, national surveillance of NTDs and other birth defects remains limited worldwide. To promote global birth defects surveillance efforts, in 2010 the World Health Assembly issued a resolution urging member states “to develop and strengthen registration and surveillance systems for birth defects” [11].

There have been recent efforts to model and estimate the worldwide burden of NTDs and other major birth defects [1, 12]. Some data are also available from systematic reviews, but most of the reviews are specific to certain regions or income levels [13–15]. However, an accurate estimate of the prevalence of NTDs in most countries is still unknown primarily due to insufficient and fragmented data collection. To complement previous efforts, the goal of our review is to describe the most current prevalence estimates of NTDs worldwide, while highlighting key methodological differences and gaps in available data.

## Methods

### Search Strategy

We followed the Preferred Reporting Items for Systematic Reviews and Meta-Analyses (PRISMA) guidelines (S1 Document) [16]. We searched the following bibliographic databases for English and Spanish language literature published between January 1990 and July 2014: the Cochrane Collaboration, CINAHL, Embase, POPLINE, PubMed, Global Health (CDC resource), Web of Science, and several World Health Organization (WHO) library resources (African Index Medicus, Index Medicus for the Eastern Mediterranean Region, Spanish Health Sciences Bibliographic Index, Index Medicus for the South-East Asian Region, Latin American and Caribbean Health Sciences Literature, and the World Health Organization Library Information System). We adapted the search terms to each database and included keywords for neural tube defects, congenital anomalies, epidemiology, registries, and hospitals. We also identified international birth defect registries and searched the databases/reports of the European Surveillance of Congenital Anomalies (EUROCAT), the International Clearinghouse for Birth Defects Surveillance and Research (ICBDSR), and other reports. Finally, we included additional studies and reports from hand searching reference lists of systematic reviews.

### Inclusion/Exclusion Criteria and Algorithm Review

We included case-control and cross-sectional studies and reports with either a reported prevalence of NTDs (defined as anencephaly/spina bifida/encephalocele), or numerator (number of reported NTD cases) and denominator data (number of births in the study population). Many studies reported on NTDs without explaining how they defined them; we included these studies in order to increase coverage.

We excluded the following: 1) case reports and supplementation trials; 2) studies that only included anencephaly and/or encephalocele; 3) studies that only counted non-NTDs per our definition, such as amniotic band sequence, chromosomal abnormalities, or spina bifida occulta; 4) studies with a denominator of fewer than 5,000 total births given the high degree of uncertainty of NTD prevalence in such a small sample size; 5) studies that reported prevalence in graphs without point estimates; 6) studies that only used mortality data; 7) studies with data based only on prenatal diagnosis; 8) and studies whose data were collected prior to 1990. We also excluded studies that reported data after a contamination event that may have caused an increase in NTD prevalence estimates.

We developed an algorithm to ensure that the most current and relevant data for each country were included in our review. If multiple studies were available for the same region or country but at different time periods, we included the study with the most recent data. In instances where multiple studies existed for one country from different geographic locations, all studies from that country were included, except if nationally representative data were available. In these cases, only the nationally representative study was used. However, if one study reported nationwide data that were not nationally representative, we still included studies from individual regions.

### Data Abstraction and Risk-of-Bias (RoB) Assessment

We abstracted data on the number of cases (numerator), the birth cohort (denominator), and calculated prevalence into a standard table. Three authors reviewed the abstracted data from the original reports and corrected errors in both abstraction and the original reports. To verify the reported prevalence estimates and to exclude syndromes, chromosomal abnormalities, isolated hydrocephalus, and spina bifida occulta cases, we re-calculated the prevalence of anencephaly, spina bifida, and encephalocele. We also calculated a sum of reported NTDs, which included spina bifida and/or anencephaly and encephalocele, depending on what NTDs the authors of the original study assessed. In addition to prevalence, we also abstracted the following information for each study: years included, geographic location, inclusion/exclusion criteria, study design (population-based vs. hospital-based), and whether the data were gathered from a birth defects registry/surveillance system. We did not distinguish between registries and surveillance systems in this review.

We developed and pre-piloted a risk-of-bias (RoB) tool to assess the quality of each study’s methodology. A study’s RoB score was based on the following components: study design, case ascertainment methods, case definition, representativeness, and limitations. The lower the RoB score, the less the study was considered to be prone to bias. Two authors reviewed each study independently and their scores were averaged for a single RoB score (possible score range: 0.0–18.0). We placed final RoB scores into quartiles: low (0.0–5.4), moderately low (5.5–7.9), moderately high (8.0–10.9), or high (11.0–18.0). We assigned the lowest RoB scores to studies that: were based on surveillance systems or registries; were population-based; were representative (as defined by the original authors to accurately describe their population of interest); included an NTD case definition; defined inclusion and exclusion criteria (e.g., gestational age, birth weight, birth outcome); and had case reporting from multiple sources.

### Analysis

As part of our analyses, we stratified countries by WHO regions, World Bank income levels (low, lower-middle, upper-middle, high), presence of a surveillance system/registry, and RoB quartiles [17, 18]. For publications that did not provide NTD prevalence, we calculated the sum of reported NTDs and individual NTD type-specific prevalence estimates. In addition, if it was not provided by the reference, we calculated the 95% confidence interval for each prevalence using the Poisson distribution if the number of cases was below 30, and using the binomial distribution if the number of cases was greater than or equal to 30. We calculated the range and median reported NTD prevalence for each WHO region.

We used ArcGIS 10.2.1 (ESRI, Redlands, California) to create maps illustrating NTD prevalence distributions and registry/surveillance coverage. On the maps, NTD prevalence was classified into quintiles based on all reported prevalence estimates. If there were national data, the entire country was filled-in. In Europe, if regional data were available, this geographical level was also filled-in. In instances where multiple prevalence estimates were available at the national level, the prevalence reported by the study/report with the least RoB was selected. Graphical representations of data were created using SigmaPlot 12.5 (Systat Software, San Jose, California).

## Results

### PRISMA

**Fig 1:**
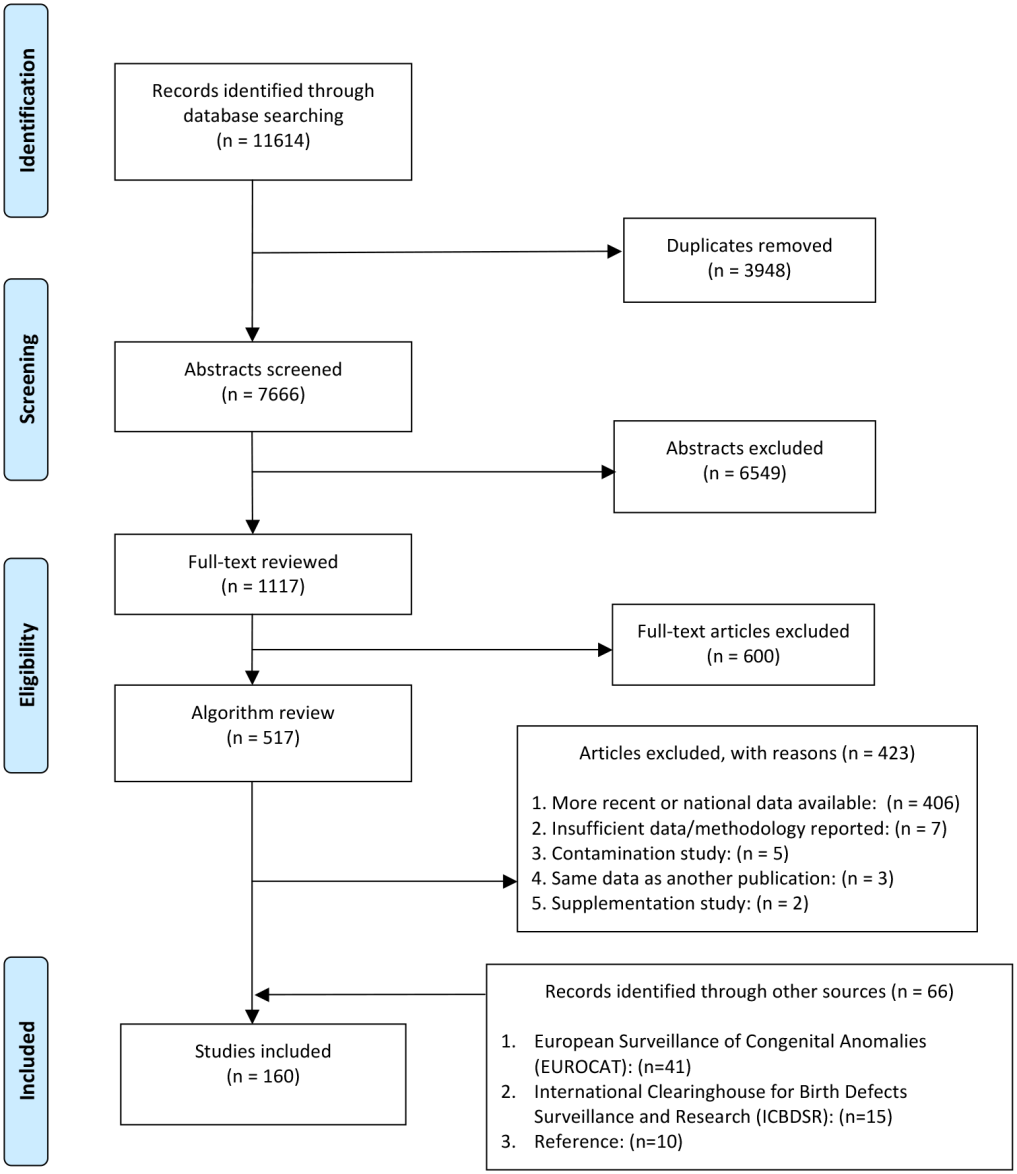
PRISMA Flowchart.

The literature search yielded 11,614 results, of which 3,948 were duplicates. Two authors reviewed and screened the 7,666 unique titles and abstracts for inclusion and exclusion criteria. After this initial screening, we excluded 6,549 abstracts and conducted the first wave of full-text review for the remaining 1,117 citations, in which 600 more were excluded. We then evaluated the remaining 517 citations and an additional 66 hand-searched sources from reports such as ICBDSR and author contacts to ensure the most relevant sources (i.e., most up-to-date data) were included. We identified 160 unique studies and reports published between January 1990 and July 2014 that met our inclusion criteria in the final stage of review (Fig 1).

The results represent data from 75 countries. Among the 194 WHO member states, the percent reporting within each region is as follows: African (8/47; 17%), Eastern Mediterranean (12/21; 57%), European (26/53; 49%), Americas (15/35; 43%), South-East Asian (4/11; 36%) and Western Pacific (9/27; 33%). Of the countries in our review, 46% have high, 31% have upper-middle, 16% have lower-middle, and 7% have low income status as defined by the World Bank.

Of the 160 studies, 2% reported spina bifida alone, 10% spina bifida and anencephaly, 1% spina bifida and encephalocele, and 81% reported all 3 conditions (either stratified or not). Six percent of studies did not provide a clear definition of how they defined NTDs.

### Prevalence of Neural Tube Defects

**Fig 2:**
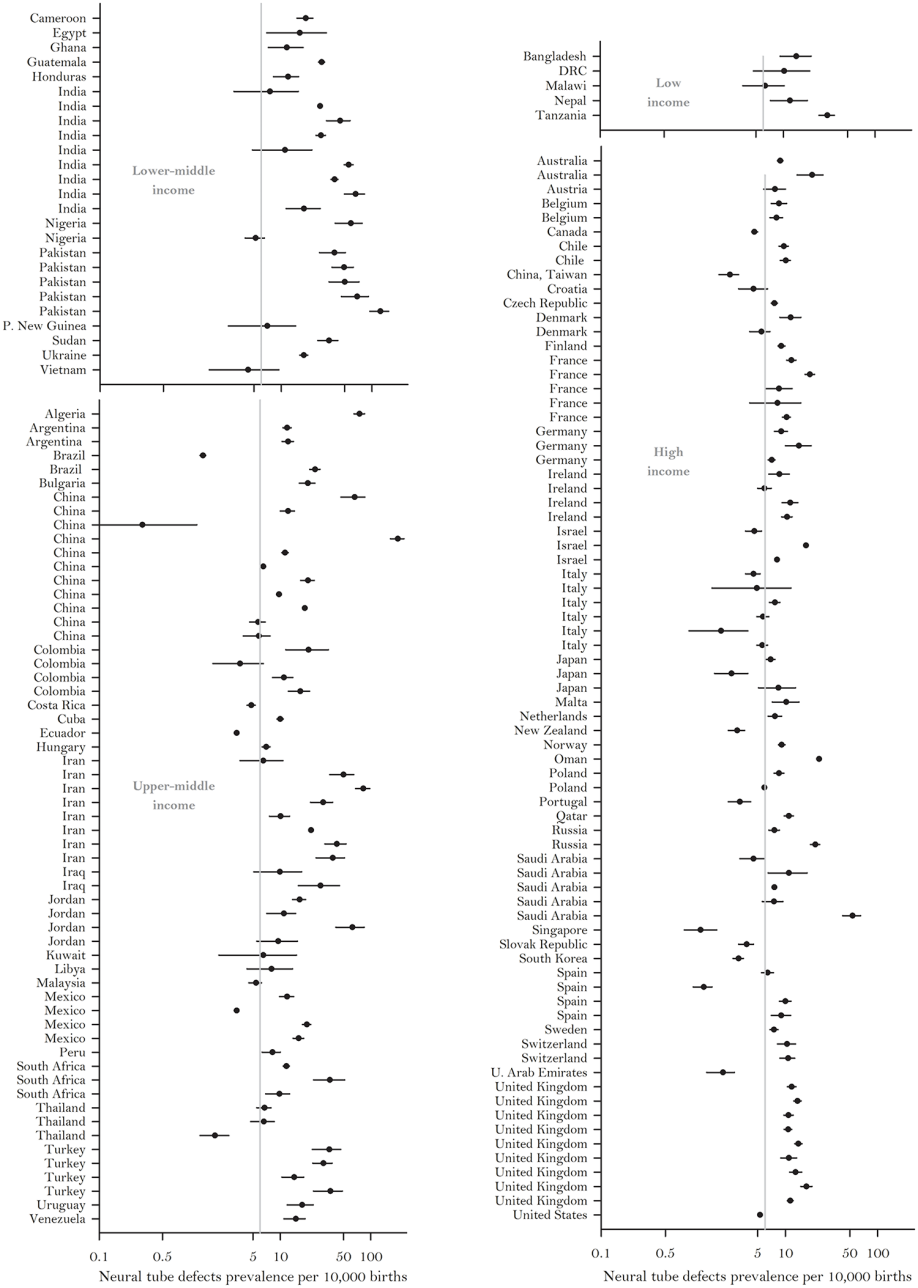
Neural Tube Defects Prevalence and Confidence Intervals by World Bank Income Classifications (Log Scale)[18].

This systematic review demonstrates great variability in reported NTD prevalence estimates globally (range: 0.3–199.4 per 10,000 births) (Table 1) [19–124]. Of note, both the lowest and highest point estimates in this global range came from studies conducted in different regions of China; Beijing [113] and Luliang [112], respectively. However, even after excluding these estimates, the global range is still quite variable (range: 1.2–124.1 per 10,000 births) (Table 1) [122, 48]. Fig 2 also illustrates that NTD prevalence estimates throughout the world are high, with approximately 80% of reported prevalence estimates above 6.0 per 10,000 births (i.e., the approximate rate that should be attainable through adequate periconceptional folic acid intake) [8].

**Table 1:** Neural Tube Defect (NTD) Prevalence Estimates by World Health Organization (WHO) Region*.

Country	World Bank Classification	Location	Author	Year(s) Included	Prevalence Rate per 10,000 Births							
					Anencephaly		Spina bifida		Encephalocele		Sum of Reported NTDs^¥^	
					Prevalence	95% CI	Prevalence	95% CI	Prevalence	95% CI	Prevalence	95% CI
**AFRICA**												
Algeria	Upper-middle	Setif	Houcher, et al.[19]	2004–2006	32.2	(25.6, 38.8)	42.8^[f^^]^	(35.2, 50.4)	0.3	(0.0, 2.0)	75.4	(65.4, 85.4)
Cameroon	Lower-middle	Yaounde	Njamnshi AK, et al.[20]	January 1997- December 2006							18.6	(14.9, 22.3)
Democratic Republic of Congo	Low	Nyankunde, Oriental Province	Ahuka OL, et al.[21]	January 1993–August 2001	1.1	(0.0, 6.3)	6.8	(2.5, 14.8)	2.3	(0.3, 8.2)	10.2	(4.7, 19.4)
Ghana	Lower-middle	Accra	Anyebuno M, et al.[22]	January 1991–December 1992	8.4	(4.8, 13.6)	3.1	(1.2, 6.8)			11.5	(7.2, 17.4)
Malawi	Low	Blantyre	Msamati BC, et al.[23]	1998–1999			6.3	(3.6, 10.2)			6.3	(3.6, 10.2)
Nigeria	Lower-middle	Cross River and Akwa Ibom States	Ekanem TB, et al.[24]	1980–2003	1.6	(1.0, 2.4)	3.7	(2.7, 4.8)			5.2	(4.0, 6.5)
Nigeria	Lower-middle	Jos	Airede KI [25]	June 1987–June 1990	3.3	(0.4, 12.1)	41.8	(27.1, 61.7)	13.4	(5.8, 26.4)	58.6^[a]^	(39.3, 78.0)
South Africa	Upper-middle	Eastern Cape, Kwazulu Natal, Mpumalanga, and Free State Provinces	Sayed AR, et al.[26]	October 2004–June 2005	3.7	(2.2, 5.9)	5.4	(3.5, 8.0)			9.8	(6.9, 12.7)
South Africa	Upper-middle	Sovenga, Northern Transvaal	Venter PA, et al.[27]	June 1989–December 1992	17.1	(9.1, 29.2)	15.8	(8.1, 27.5)	2.6	(0.3, 9.5)	35.4	(23.4, 51.6)
South Africa	Upper-middle	Cape Town	Viljoen DL, et al. [28]	1973–1992							11.7	(10.8, 12.6)
Tanzania	Low	Dar es Salaam	Kinasha AD and Manji K [29]	2000–2002	1.2	(0.3, 3.0)	26.1	(20.7, 31.5)	2.9	(1.4, 5.4)	30.2	(24.4, 36.0)
**EASTERN MEDITERRANEAN**												
Egypt	Lower-middle	Upper Egypt	Mohammed YA, et al.[30]	March 2007–October 2007	2.0	(0.1, 11.1)	10.0	(3.3, 23.3)	4.0	(0.5, 14.5)	16.0^[b^^]^	(6.9, 31.5)
Iran	Upper-middle	Yasuj, South West Iran	Ebrahimi S, et al.[31]	March 2008–February 2011							38.1	(24.9, 51.3)
Iran	Upper-middle	Ahvaz	Behrooz AG and Gorjizadeh MH [32]	March 2002–March 2004	24.9	(16.4, 33.4)	15.1	(9.2, 23.3)	2.3	(0.5, 6.6)	42.2	(31.2, 53.3)
Iran	Upper-middle	Gorgan, Golestan	Abdollahi Z, et al.[33]	December 2007–December 2008							21.9	N/A
Iran	Upper-middle	Tehran	Delshad S, et al.[34]	March 2005-March 2007			8.5	(6.2, 10.8)	1.6	(0.8, 3.0)	10.1	(7.6, 12.7)
Iran	Upper-middle	Birjand	Afshar M, et al.[35]	April 1997–December 2001	15.5	(10.1, 22.7)	8.9	(5.0, 14.7)	1.8	(0.4, 5.2)	29.8	(21.6, 38.0)
Iran	Upper-middle	Urmia	Rad IA, et al.[36]	January 2001–June 2005	55.2	(43.0, 67.5)	24.8	(16.6, 33.0)	2.8	(0.8, 7.3)	82.9	(67.9, 97.8)
Iran	Upper-middle	Hamadan Province	Farhud DD, et al.[37]	1991–1997	15.6	(8.1, 25.9)	7.0	(2.6, 15.2)			50.1	(35.2, 65.0)
Iran	Upper-middle	Tabriz	ICBDSR 2011 Report [38]	2009	4.7	(2.4, 8.5)	0.9	(0.1, 3.1)	0.9	(0.1, 3.1)	6.5	(3.6, 10.7)
Iraq	Upper-middle	Al-Ramadi, Al-Anbar Governate	Al-Ani ZR, et al.[39]	October 2010 –October 2011	3.5	(0.4, 12.6)	15.7	(7.2, 29.8)	8.7	(2.8, 20.3)	27.9^[a^^]^	(15.9, 45.2)
Iraq	Upper-middle	Basrah	Al-Sadoon I, et al.[40]	1990	2.5	(0.5, 7.2)	7.4	(3.4, 14.1)			9.9	(5.1, 17.2)
Jordan	Upper-middle	North Jordan	Amarin ZO and Obeidat AZ [41]	2005–2006							9.5	(5.5, 15.5)
Jordan	Upper-middle	Amman	Aqrabawi HE [42]	April 2002 –April 2003	0.0	(0.0, 7.3)	59.0	(37.9, 80.0)	3.9	(0.5, 14.2)	62.9^[a^^]^	(41.2, 84.6)
Jordan	Upper-middle	Amman	Masri AT [43]	1993–2002	3.5^[c^^]^	(1.7, 6.5)	7.1^[c^^]^	(4.3, 10.9)	0.4^[c^^]^	(0.0, 2.0)	11.0^[c^^]^	(7.1, 14.8)
Jordan	Upper-middle	Irbid Province	Daoud AS, et al.[44]	January 1991–December 1993	3.7	(2.4, 5.0)	10.0	(7.9, 12.1)	2.6	(1.7, 4.0)	16.4	(13.7, 19.1)
Kuwait	Upper-middle	Al-Jahara Region	Madi SA, et al.[45]	January 2000–December 2001	3.9	(0.8, 11.3)	2.6	(0.3, 9.3)			6.5	(2.1, 15.1)
Libya	Upper-middle	Benghazi	Singh R and Al-Sudani O [46]	1995	7.4	(3.8, 13.0)	0.6	(0.0, 3.4)			8.0	(4.3, 13.7)
Oman	High	National	Alasfoor D and ElSayed MK [47]	2010			6.8	N/A			23.2	N/A
Pakistan	Lower-middle	Swat	Khattak ST, et al.[48]	Januray 2007–December 2007	113.3	(85.5, 141.1)	7.2	(2.0, 18.4)			124.1^[d]^	(95.0, 153.2)
Pakistan	Lower-middle	Peshawar	Qazi G [49]	Januray 2009–December 2009	47.2	(30.3, 70.2)	21.6	(10.8, 38.7)			68.8	(46.1, 91.6)
Pakistan	Lower-middle	Karachi	Perveen F and Tyyab S [50]	January 2000–October 2005	29.4	(17.2, 47.1)	15.6	(7.1, 29.6)	5.2	(1.1, 15.2)	50.2	(33.6, 72.1)
Pakistan	Lower-middle	Lahore	Najmi RS [51]	November 1994–October 1996; August 1997–March 1998	29.6	(19.5, 39.7)	17.0	(10.3, 26.6)	2.7	(0.6, 7.9)	49.3	(36.3, 62.3)
Pakistan	Lower-middle	Karachi	Jooma R [52]	2002	19.8	(11.6, 30.0)	15.7	(8.5, 25.0)	3.1	(0.6, 8.9)	38.6	(26.4, 50.9)
Qatar	High	Doha	Bener A, et al.[53]	January 1985–December 2009	3.6	(2.9, 4.3)	7.3	(6.4, 8.4)			10.9	(9.7, 12.2)
Saudi Arabia	High	Al-Khobar	Al-Jama F, et al.[54]	January 1992–December 1997	22.4	(14.8, 30.0)	25.7	(17.5, 33.9)	5.4	(2.3, 10.7)	53.5	(41.7, 65.3)
Saudi Arabia	High	Asir Region	Asindi A and Al-Shehri A.[55]	January 1995–December 1998	0.4	(0.1, 1.1)	5.6	(4.0, 7.2)	1.6^[a^^]^	(0.8, 2.7)	7.5^[a^^]^	(5.6, 9.4)
Saudi Arabia	High	Jeddah	Safdar OY, et al.[56]	2001–2005							7.6	N/A
Saudi Arabia	High	Al-Madinah Al-Munawarah	Murshid WR [57]	April 1996–March 1997			10.9	(6.5, 17.2)			10.9	(6.5, 17.2)
Saudi Arabia	High	Riyadh	Hakami WS and Majeed-Saidan MA [58]	January 2001–December 2010							4.5	(3.2, 5.9)
Sudan	Lower-middle	Omdurman	Elsheikh GEA and Ibrahim SA [59]	February 2003–January 2004	12.5	(7.4, 17.6)	16.3	(10.5, 22.1)	4.9	(2.2, 9.3)	33.7^[a^^]^	(25.3, 42.1)
United Arab Emirates	High	National	Al Hosani H, et al.[60]	January 1999–December 2001							2.1^[f^^]^	(1.4, 2.8)
**EUROPE**												
Austria	High	Styria	EUROCAT [61]	2003–2009	1.7	(0.9, 2.9)	4.6	(3.1, 6.4)	1.5	(0.8, 2.7)	7.7	(5.8, 10.0)
Belgium	High	Antwerp	EUROCAT [61]	2003–2012	2.6	(2.0, 3.5)	4.5	(3.6, 5.5)	0.8	(0.5, 1.4)	8.0	(6.8, 9.3)
Belgium	High	Hainaut	EUROCAT [61]	2003–2012	3.2	(2.3, 4.4)	4.1	(3.1, 5.4)	1.2	(0.7, 2.0)	8.5	(7.0, 10.3)
Bulgaria	Upper-middle	Plevin Region	Kovacheva K, et al.[62]	1988–2006							20.2	(16.2, 24.2)
Croatia	High	Zagreb	EUROCAT [61]	2003–2012	2.0	(1.1, 3.3)	1.4	(0.7, 2.6)	1.1	(0.5, 2.2)	4.5	(3.1, 6.4)
Czech Republic	High	National	EUROCAT [61]	2003–2010	2.4	(2.1, 2.8)	3.9	(3.5, 4.3)	1.3	(1.0, 1.5)	7.6	(7.0, 8.2)
Denmark	High	National	Pasternak B, et al.[63]	1997–2011							5.5	(4.1, 6.8)
Denmark	High	Odense	EUROCAT [61]	2003–2012	4.1	(2.5, 6.2)	5.8	(3.9, 8.3)	1.5	(0.7, 3.1)	11.4	(8.7, 14.7)
Finland	High	National	EUROCAT [61]	2003–2011	3.2	(2.7, 3.7)	4.0	(3.5, 4.6)	1.9	(1.5, 2.3)	9.0	(8.3, 9.9)
France	High	Bas-Rhin	Stoll C, et al.[64]	1979–2008	4.3^[a^^]^	(3.7, 4.9)	4.8^[a^^]^	(4.1, 5.5)	1.2^[a^^]^	(0.9, 1.5)	10.3^[a^^]^	(9.3, 11.3)
France	High	Auvergne	EUROCAT [61]	2002	2.2	(0.4, 6.6)	3.0	(0.8, 7.7)	3.0	(0.8, 7.7)	8.2	(4.1, 14.7)
France	High	French West Indies	EUROCAT [61]	2009–2012	3.3	(1.8, 5.6)	4.0	(2.3, 6.5)	1.2	(0.4, 2.8)	8.5	(6.0, 11.8)
France	High	Ile de la Reunion	EUROCAT [61]	2003–2012	7.3	(6.0, 8.8)	9.1	(7.6, 10.8)	2.0	(1.3, 2.9)	18.4	(16.3, 20.7)
France	High	Paris	EUROCAT [61]	2003–2012	4.7	(3.9, 5.6)	5.1	(4.3, 6.1)	1.8	(1.3, 2.4)	11.6	(10.3, 13.0)
Germany	High	Northern Rhine Region	Klusmann A, et al.[65]	January 1996 -December 2003	1.9	(1.6, 2.2)	4.4	(3.9, 4.9)	0.8	(0.6, 1.0)	7.1	(6.5, 7.7)
Germany	High	Mainz	EUROCAT [61]	2003–2011	3.8	(1.9, 6.9)	6.6	(4.0, 10.4)	3.5	(1.7, 6.4)	14.0	(10.0, 19.0)
Germany	High	Saxony-Anhalt	EUROCAT [61]	2003–2012	2.0	(1.4, 2.8)	5.6	(4.6, 6.9)	1.4	(0.9, 2.1)	9.0	(7.6, 10.5)
Hungary	Upper-middle	National	ICBDSR 2011 Report [38]	2005–2009	2.0	(1.6, 2.4)	4.4	(3.8, 5.0)	0.6	(0.4, 0.9)	7.0	(6.3, 7.7)
Ireland	High	National	McDonnell R, et al.[66]	2009–2011	4.7	(3.8, 5.6)	5.1	(4.2, 6.0)	0.7	(0.4, 1.1)	10.4	(9.1, 11.8)
Ireland	High	Cork & Kerry	EUROCAT [61]	2003–2012	4.9	(3.6, 6.5)	5.4	(4.0, 7.0)	1.0	(0.5, 1.9)	11.3	(9.2, 13.6)
Ireland	High	Dublin	EUROCAT [61]	2003–2012	2.2	(1.7, 2.9)	3.0	(2.4, 3.8)	0.7	(0.4, 1.1)	5.9	(5.0, 7.0)
Ireland	High	South East Ireland	EUROCAT [61]	2003–2012	3.3	(2.1, 4.9)	5.0	(3.6, 6.9)	0.3	(0.0, 1.0)	8.6	(6.6, 11.0)
Israel	High	National	Zlotogora J, et al.[67]	2002–2004								
		Jews			4.9	N/A	2.7	N/A			8.1	N/A
		Arabs and Druze			8.2	N/A	6.2	N/A			16.7	N/A
Israel	High	Multi-Regional	ICBDSR 2011 Report [38]	2005–2009	1.3	(0.8, 1.8)	2.9	(2.2, 3.6)	0.5	(0.2, 0.9)	4.6	(3.7, 5.5)
Italy	High	Emilia Romagna	EUROCAT [61]	2003–2012	2.2	(1.7, 2.7)	2.7	(2.2, 3.3)	0.7	(0.5, 1.0)	5.6	(4.9, 6.4)
Italy	High	Sicily	EUROCAT [61]	2003–2004	0.5	(0.1, 1.8)	1.5	(0.5, 3.3)	0.0	(0.0, 0.9)	2.0	(0.9, 3.9)
Italy	High	Tuscany	EUROCAT [61]	2003–2012	1.9	(1.5, 2.5)	3.1	(2.5, 3.8)	0.7	(0.4, 1.1)	5.7	(4.9, 6.6)
Italy	High	Campania	ICBDSR 2011 Report [38]	2005–2009	3.6	(2.9, 4.2)	3.1	(2.5, 3.8)	1.0	(0.6, 1.3)	7.7	(6.7, 8.7)
Italy	High	Lombardy	ICBDSR 2011 Report [38]	2009	2.0	(0.2, 7.1)	2.0	(0.2, 7.1)	1.0	(0.0, 5.5)	4.9	(1.6, 11.5)
Italy	High	North East Italy	ICBDSR 2011 Report [38]	2005–2009	1.5	(1.0, 2.0)	2.5	(1.9, 3.1)	0.5	(0.2, 0.8)	4.5	(3.7, 5.3)
Malta	High	National	EUROCAT [61]	2003–2011	2.2	(0.9, 4.3)	6.3	(4.0, 9.5)	1.6	(0.6, 3.6)	10.2	(7.2, 14.0)
Netherlands	High	Northern Netherlands	EUROCAT [61]	2003–2012	2.6	(1.9, 3.5)	4.6	(3.7, 5.7)	0.6	(0.3, 1.0)	7.7	(6.5, 9.1)
Norway	High	National	EUROCAT [61]	2003–2012	3.5	(3.0, 4.0)	4.7	(4.1, 5.2)	0.9	(0.7, 1.2)	9.1	(8.4, 9.9)
Poland	High	National	EUROCAT [61]	2003–2010	0.8	(0.7, 0.9)	4.5	(4.3, 4.8)	0.6	(0.5, 0.7)	5.9	(5.7, 6.2)
Poland	High	Wielkopolska	EUROCAT [61]	2003–2010	1.2	(0.8, 1.7)	6.3	(5.5, 7.3)	1.0	(0.7, 1.4)	8.5	(7.5, 9.6)
Portugal	High	South Portugal	EUROCAT [61]	2003–2011	1.2	(0.8, 1.9)	1.8	(1.2, 2.5)	0.2	(0.1, 0.6)	3.2	(2.4, 4.2)
Russia	High	Arkhangelskaja Oblast	Petrova JG and Vaktskjold A [68]	1995–2004	10.7	(9.0, 12.4)	10.4	(8.7, 12.1)			21.1	(18.7, 23.5)
Russia	High	Moscow	ICBDSR 2011 Report [38]	2005–2009	2.9	(2.3, 3.5)	3.7	(3.0, 4.4)	1.1	(0.7, 1.4)	7.6	(6.6, 8.6)
Slovak Republic	High	Multi-Regional	ICBDSR 2011 Report [38]	2005–2009	0.9	(0.6, 1.3)	2.2	(1.7, 2.8)	0.7	(0.4, 1.0)	3.8	(3.1, 4.5)
Spain	High	Barcelona	EUROCAT [61]	2003–2007	4.9	(3.4, 6.8)	3.3	(2.1, 4.9)	0.8	(0.3, 1.8)	9.0	(7.0, 11.4)
Spain	High	Basque Country	EUROCAT [61]	2003–2011	5.2	(4.2, 6.3)	4.1	(3.2, 5.2)	0.7	(0.4, 1.2)	10.0	(8.6, 11.5)
Spain	High	National	EUROCAT [61]	2003–2012	0.3	(0.2, 0.5)	0.9	(0.6, 1.1)	0.2	(0.1, 0.3)	1.3	(1.0, 1.6)
Spain	High	Valencia Region	EUROCAT [61]	2007–2011	2.4	(1.9, 3.1)	2.4	(1.9, 3.1)	1.5	(1.1, 2.1)	6.4	(5.5, 7.4)
Sweden	High	National	EUROCAT [61]	2007–2011	2.8	(2.4, 3.3)	3.8	(3.3, 4.3)	1.0	(0.7, 1.3)	7.5	(6.8, 8.3)
Switzerland	High	National	Poretti A, et al.[69]	January 2001–December 2007	1.8^[a, b]^	(1.0, 2.6)	7.8^[a]^	(6.1, 9.5)	1.1^[a]^	(0.6, 2.0)	10.7^[a]^	(8.7, 12.6)
Switzerland	High	Vaud	EUROCAT [61]	2003–2012	3.5	(2.3, 5.2)	4.5	(3.1, 6.2)	2.4	(1.4, 3.7)	10.4	(8.2, 12.9)
Turkey	Upper-middle	Afyonkarahisar	Onrat ST, et al.[70]	July 2003–December 2004	13.9	(7.2, 24.3)	19.7	(11.5, 31.5)	2.3	(0.3, 8.4)	35.9	(23.3, 48.5)
Turkey	Upper-middle	Izmir	Mandiracioglu A, et al.[71]	January 2000–December 2000							14.3^[a, b]^	(10.4, 18.2)
Turkey	Upper-middle	Multi-Regional	Tuncbilek E, et al.[72]	July 1993–June 1994	11.0	(7.0, 16.3)	13.2	(8.4, 18.0)	5.9	(3.2, 10.2)	30.1	(22.9, 37.4)
Turkey	Upper-middle	Ankara	Himmetoglu O, et al.[73]	1988–1995							34.9	(22.6, 46.6)
Ukraine	Lower-middle	Rivne and Khmelnytsky Provinces^[g]^	EUROCAT [61]	2005–2011	7.0	(5.9, 8.2)	9.0	(7.8, 10.4)	1.7	(1.2, 2.4)	17.7	(16.0, 19.6)
United Kingdom	High	East Midlands and South Yorkshire	EUROCAT [61]	2003–2012	4.9	(4.4, 5.5)	5.3	(4.8, 5.9)	1.0	(0.8, 1.3)	11.3	(10.5, 12.1)
United Kingdom	High	Glasgow	EUROCAT [61]	1990–2000	6.8	(5.4, 8.4)	7.8	(6.3, 9.6)	2.4	(1.6, 3.4)	16.9	(14.7, 19.4)
United Kingdom	High	Merseyside and Chesire	EUROCAT [61]	1995–1999	5.4	(4.2, 6.7)	6.5	(5.2, 8.0)	1.1	(0.6, 1.8)	12.9	(11.1, 15.0)
United Kingdom	High	North West Thames	EUROCAT [61]	2003–2004	5.0	(3.7, 6.6)	4.7	(3.4, 6.3)	1.2	(0.6, 2.1)	10.9	(8.9, 13.2)
United Kingdom	High	Northern England	EUROCAT [61]	2003–2012	5.8	(5.0, 6.6)	6.5	(5.6, 7.4)	1.6	(1.2, 2.1)	13.8	(12.6, 15.1)
United Kingdom	High	South West England	EUROCAT [61]	2005–2012	4.2	(3.6, 4.9)	5.2	(4.5, 6.0)	1.2	(0.9, 1.6)	10.7	(9.7, 11.7)
United Kingdom	High	Thames Valley	EUROCAT [61]	2003–2012	4.9	(4.1, 5.8)	4.8	(4.0, 5.8)	1.1	(0.7, 1.6)	10.8	(9.6, 12.1)
United Kingdom	High	Wales	EUROCAT [61]	2003–2012	5.1	(4.4, 5.9)	6.4	(5.6, 7.3)	2.0	(1.5, 2.5)	13.5	(12.3, 14.8)
United Kingdom	High	Wessex	EUROCAT [61]	2003–2012	5.9	(5.1, 6.9)	4.8	(4.0, 5.7)	1.0	(0.6, 1.4)	11.7	(10.5, 13.0)
**AMERICAS**												
Argentina	Upper-middle	National	Groisman B, et al.[74]	November 2009–June 2012	3.6	(2.9, 4.3)	6.4	(5.5, 7.7)	1.9	(1.5, 2.5)	11.9	(10.7, 13.2)
Argentina	Upper-middle	Multi-Regional	Lopez-Camelo JS, et al.[75]	2005–2007	3.7	(2.7, 4.6)	6.6	(5.3, 7.9)	2.0	(1.3, 2.8)	12.2	(10.5, 14.0)
Brazil	Upper-middle	National	Orioli IM, et al.[76]	2006			1.4	(1.3, 1.5)			1.4	(1.3, 1.5)
Brazil	Upper-middle	Multi-Regional	Lopez-Camelo JS, et al. [75]	July 2005–December 2007	6.9	(5.2, 8.6)	14.2	(11.8, 16.6)	3.2	(2.1, 4.4)	24.3	(21.2, 27.5)
Canada	High	National	ICBDSR 2011 Report [38]	2005–2009	1.0	(0.9, 1.2)	3.0	(2.7, 3.2)	0.7	(0.6, 0.8)	4.6	(4.3, 5.0)
Chile	High	Bio Bio, Los Lagos, Los Rios, Maule, Santiago Metropolitan, O'Higgins, Tarapaca, and Valparaiso Regions	Nazer J and Cifuentes L [77]	2001–2010	3.7	(3.0, 4.4)	4.5	(3.7, 5.3)	1.7	(1.2, 2.1)	9.6	(8.5, 10.7)
Chile	High	Multi-Regional	Lopez-Camelo JS, et al.[75]	2001–2007	3.7	(2.9, 4.4)	4.6	(3.8, 5.5)	1.8	(1.3, 2.3)	10.1	(8.8, 11.3)
Colombia	Upper-middle	Cali	Pachajoa H, et al.[78]	March 2004–October 2008	6.4	(3.9, 9.7)	7.3	(4.7, 10.8)	3.0	(1.5, 5.6)	16.7	(12.3, 21.1)
Colombia	Upper-middle	Bogota, Ubate, and Manizales	Zarante I, et al.[79]	April 2001–January 2008							11.0	(8.2, 13.8)
Colombia	Upper-middle	Bogota	ICBDSR 2011 Report [38]	2009	1.6	(0.5, 3.8)	2.0	(0.7, 4.3)	0.0	(0.0, 1.2)	3.6	(1.8, 6.5)
Colombia	Upper-middle	Baraya, Garzon, Neiva, and Palermo	Ostos H, et al.[80]	1998	9.6	(3.9, 19.8)	9.6	(3.9, 19.8)	1.4	(0.0, 7.7)	20.6	(11.5, 34.0)
Costa Rica	Upper-middle	National	de la Paz Barboza-Arguello M, et al.[81]	2003–2012							4.8	(4.3, 5.3)
Cuba	Upper-middle	National	ICBDSR 2011 Report [38]	2005–2009	3.8	(3.3, 4.3)	4.4	(3.9, 5.0)	1.7	(1.4, 2.1)	10.0	(9.2, 10.8)
Ecuador	Upper-middle	Multi-Regional	Gonzalez-Andrade F and Lopez-Pulles R [82]	2001–2007	0.3	(0.3, 0.4)	2.5	(2.3, 2.7)	0.5	(0.4, 0.6)	3.3	(3.1, 3.5)
Guatemala	Lower-middle	National	Acevedo CR, et al.[83]	2001–2003	2.3	(1.7, 2.9)	22.7	(20.8, 24.6)	3.0	(2.3, 3.7)	27.9	(25.8, 30.0)
Honduras	Lower-middle	Tegucigalpa	Hernandez R and Alvarenga R [84]	July 1998–September 2000							11.9	(8.2, 15.5)
Mexico	Upper-middle	Monterrey, Nuevo Leon	Hernandez-Herrera RJ, et al.[85]	1995–1999	6.5	(5.1, 7.9)	8.2	(6.6, 9.7)	1.3	(0.8, 2.1)	16.0	(13.9, 18.2)
Mexico	Upper-middle	Guadalajara	Alfaro N, et al.[86]	1988–1999	9.5	(8.0, 10.9)	10.3	(8.8, 11.8)			19.7	(17.6, 21.8)
Mexico	Upper-middle	National	Navarrete Hernandez E, et al.[87]	2009–2010	2.1	(1.9, 2.2)	1.2	(1.1, 1.3)			3.3	(3.1, 3.5)
Mexico	Upper-middle	National	ICBDSR 2011 Report [38]	2005–2009	4.6	(3.3, 5.9)	5.8	(4.3, 7.2)	1.6	(0.9, 2.5)	11.9	(9.8, 14.1)
Peru	Upper-middle	Lima	Sanabria Rojas HA, et al.[88]	2006–2010	1.9	(1.1, 3.1)	6.1^[a^^]^	(4.5, 7.8)	0.1	(0, 0.6)	8.2^[a^^]^	(6.3, 10.0)
Uruguay	Upper-middle	Montevideo	Castilla EE, et al.[89]	1999–2001							17.5	(11.9, 23.1)
United States	High	National	Canfield MA, et al.[90]	1999–2007	1.3	(1.2, 1.4)	3.2	(3.1, 3.3)	0.8	(0.7, 0.8)	5.3	(5.1, 5.4)
Venezuela	Upper-middle	Maracaibo, Coro, and Ciudad Bolivar	Castilla EE, et al.[89]	2000–2001							14.9	(11.0, 18.8)
**SOUTH-EAST ASIA**												
Bangladesh	Low	Dhaka	Dey AC, et al.[91]	August 2006–July 2007							13.8	(9.2, 20.0)
India	Lower-middle	Kolkata	Sarkar S, et al.[92]	September 2011–August 2012	1.6	(0.2, 5.6)	14.0	(8.3, 22.1)	2.3	(0.5, 6.8)	17.8	(11.3, 26.8)
India	Lower-middle	Delhi	Sood M, et al.[93]	January 1988–August 1990	39.0	(26.3, 51.8)	26.0	(16.7, 38.7)	1.1	(0.0, 6.0)	66.2	(49.7, 82.8)
India	Lower-middle	Lucknow	Sharma AK, et al.[94]	1982–1991	19.2	(16.8, 21.6)	19.6^[e^^]^	(17.2, 22.0)			38.8^[d^^]^	(35.4, 42.2)
India	Lower-middle	Pondicherry	Mahadevan B and Bhat BV [95]	July 1998–June 2004	18.0	(14.5, 21.6)	31.0	(26.3, 35.7)	7.0	(4.8, 9.2)	55.5^[a^^]^	(49.3, 61.8)
India	Lower-middle	Duragpur	Duttachoudhury A and Pal SK [96]	January 1991 -December 1993	5.5	(1.5, 14.1)	5.5	(1.5, 14.1)			11.0	(4.8, 21.8)
India	Lower-middle	Erode	Ponne S and Lakshmi UK [97]	2000–2004	10.7	(6.6, 12.7)	14.7	(12.3, 17.2)	1.9	(1.1, 2.8)	27.4	(24.1, 30.7)
India	Lower-middle	Himachal Pradesh Shimla	Grover N [98]	January 1991–December 1995	20.8	(12.9, 31.8)	16.8	(9.8, 27.0)	6.9	(2.8, 14.3)	44.6	(31.6, 57.5)
India	Lower-middle	Multi-Regional	ICBDSR 2011 Report [38]	2005–2009	12.3	(11.4, 13.1)	11.0	(10.2, 11.8)	3.6	(3.1, 4.0)	26.8	(25.6, 28.1)
India	Lower-middle	Sevagram, Wardha	Taksande A, et al.[99]	January 2005–July 2007			5.3	(1.7, 12.4)	2.1	(0.3, 7.7)	7.5	(3.0, 15.4)
Nepal	Low	Thapathali	Malla BK [100]	2004	5.3	(2.4, 10.1)	4.7	(2.0, 9.3)	1.8	(0.4, 5.2)	11.8	(7.2, 18.2)
Thailand	Upper-middle	Songkhla, Phatthalung, and Trang Provinces	Jaruratanasirikul S, et al.[101]	January 2001–December 2012	0.8	(0.4, 1.4)	0.7	(0.4, 1.3)	0.3	(0.1, 0.8)	1.9	(1.3, 2.7)
Thailand	Upper-middle	Chiang Mai	Kitisomprayoonkul N and Tongsong T [102]	June 1989–May 2000	5.6	(3.9, 7.4)	0.6	(0.2, 1.5)	0.4	(0.1, 1.3)	6.6	(4.7, 8.6)
Thailand	Upper-middle	Bangkok	Wasant P and Sathienkijkanchai A [103]	1990–1999	2.6	(1.8, 3.4)	3.2	(2.4, 4.1)	0.8	(0.5, 1.4)	6.7^[b, d]^	(5.5, 7.9)
**WESTERN PACIFIC**												
Australia	High	South Australia	Flood L, et al.[104]	2010							19.5	(13.4, 25.6)
Australia	High	Victoria, West Australia, South Australia, New South Wales, Queensland States	Macaldowie A and Hilder L [105]	2006–2008							8.8	(8.2, 9.4)
China	Upper-middle	Hainan Province	Fan L, et al.[106]	2010							5.8	(3.9, 7.7)
China	Upper-middle	Shenzhen City	Yang M, et al.[107]	2003–2009							5.7	(4.6, 6.8)
China	Upper-middle	National	Li X, et al.[108]	2006–2008	5.9	(5.6, 6.2)	6.0	(5.7, 6.3)	2.2	(2.0, 2.3)	14.0	(13.4, 14.5)
		Northern China			6.8	(6.4, 7.3)	9.2	(8.6, 9.8)	2.7	(2.4, 3.0)	18.7	(17.9, 19.5)
		Southern China			5.0	(4.6, 5.4)	3.1	(2.8, 3.4)	1.7	(1.5, 1.9)	9.7	(9.1, 10.3)
China	Upper-middle	Inner Mongolia	Zhang X, et al.[109]	2005–2008	6.9	(4.8, 9.0)	10.6	(8.1, 13.2)	2.7	(1.4, 4.0)	20.3^[f^^]^	(16.8, 23.8)
China	Upper-middle	National	Dai L, et al. [110]	2009							6.5	(6.1, 6.9)
China	Upper-middle	Zhejiang Province	Zhang XH, et al.[111]	2007–2009	6.3	(5.7, 7.0)	3.6	(3.1, 4.1)	1.4	(1.1, 1.7)	11.3	(10.4, 12.2)
China	Upper-middle	Luliang Prefecture, Shanxi Province	Chen G, et al.[112]	2004–2005	82.6	(60.5, 104.7)	38.9	(25.2, 57.5)	26.5	(15.4, 42.4)	199.4^[d]^	(165.2, 233.6)
China	Upper-middle	Beijing	Li Y, et al.[113]	January 2003–March 2009	0.0	(0.0, 0.6)	0.3	(0.0, 1.2)			0.3	(0.0, 1.2)
China	Upper-middle	Guizhou Province	Liu J, et al. [114]	Januray 1996–December 2004	4.2	(2.9, 5.5)	5.9	(4.4, 7.4)	0.7	(0.3, 1.4)	12.2^[d^^]^	(10.0, 14.4)
China	Upper-middle	Gansu Province	Cheng N, et al.[115]	January 2001–January 2002							66.5	(46.9, 86.1)
China	High	Taiwan	Chen BY, et al.[116]	2002	1.1	(0.7, 1.6)	1.1	(0.7, 1.6)	0.4	(0.2, 0.7)	2.5	(1.9, 3.1)
Japan	High	Osaka City	Imaizumi Y, et al.[117]	1981–1990	7.1	(4.2, 11.4)	1.3	(0.3, 3.7)			8.4	(5.1, 12.9)
Japan	High	Ishikawa Prefecture	Seto T, et al.[118]	1981–2000	0.8	(0.2, 1.3)	0.9	(0.3, 1.5)	1.0	(0.3, 1.6)	2.6	(1.7, 3.9)
Japan	High	National	ICBDSR 2011 Report [38]	2005–2009	0.9	(0.6, 1.2)	5.2	(4.5, 5.9)	0.8	(0.5, 1.1)	6.9	(6.1, 7.7)
South Korea	High	National	Kim MA, et al.[119]	2005–2006	0.2	(0.1, 0.3)	2.6	(2.2, 2.9)	0.3	(0.2, 0.4)	3.1	(2.7, 3.5)
Malaysia	Upper-middle	National	Boo NY, et al.[120]	2009	2.1	(1.5, 2.6)	1.6	(1.1, 2.1)	0.8	(0.5, 1.2)	5.4	(4.5, 6.2)
New Zealand	High	National	ICBDSR 2011 Report [38]	2005–2009	0.4	(0.2, 0.6)	2.1	(1.6, 2.6)	0.5	(0.3, 0.8)	3.0	(2.4, 3.6)
Papua New Guinea	Lower-middle	Port Moresby	Dryden R [121]	1985–1986	3.0	(0.6, 8.8)	4.0	(1.1, 10.2)			7.0	(2.6, 14.4)
Singapore	High	National	Shi LM, et al.[122]	1994–1998	0.5^[b^^]^	(0.3, 0.9)	0.7	(0.4, 1.1)			1.2^[b^^]^	(0.8, 1.8)
Vietnam	Lower-middle	Binh Thuan Province	Hoang T, et al.[123]	2010	3.6	(1.2, 8.4)	0.0	(0.0, 2.6)	0.7	(0.0, 4.0)	4.3	(1.6, 9.4)
**UNCLASSIFIED**												
Palestine		East Jerusalem and Southern West Bank	Dudin A [124]	1986–1993							54.9^[a^^]^	(46.1, 63.7)

^a^ Non-NTDs such as syndromes, chromosomal abnormalities, and spina bifida occulta were not included in our calculations

^b^ May include non-NTDs, but could not stratify in our calculation

^c^ Referred cases were not included in our calculation

^d^ Individual NTDs do not sum to total NTDs (e.g., only isolated NTD counts were provided, but prevalence includes multiple NTDs)

^e^ Spina bifida cases included encephalocele

^f^ Recalculated NTD prevalence was inconsistent with the original authors’ published rate

^g^ Regions may be impacted by Chernobyl disaster

N/A = Not applicable

* If prevalence cell is blank, data were either not reported, not stratified by specific type of NTD, or unclear

^¥^ Sum of all NTDs reported, which includes spina bifida and/or anencephaly and encephalocele, depending on what NTDs the authors of the original study assessed

**Fig 3:**
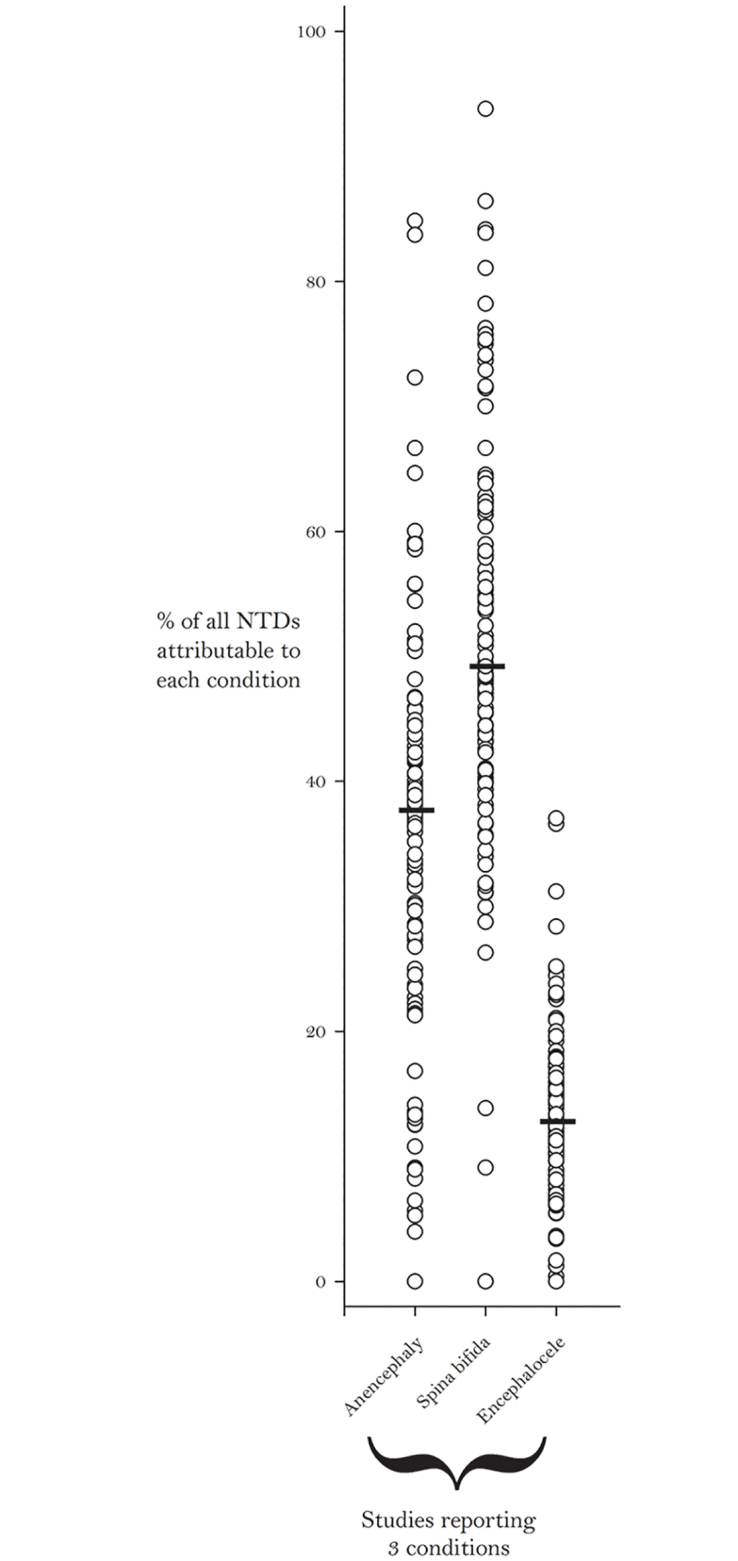
Percent of all Neural Tube Defects (NTDs) Attributable to Each Condition for Studies that Reported all Three Types of NTDs: Anencephaly, Spina Bifida, and Encephalocele. Bars Indicate the Median Percent for Each Condition.

**Fig 4:**
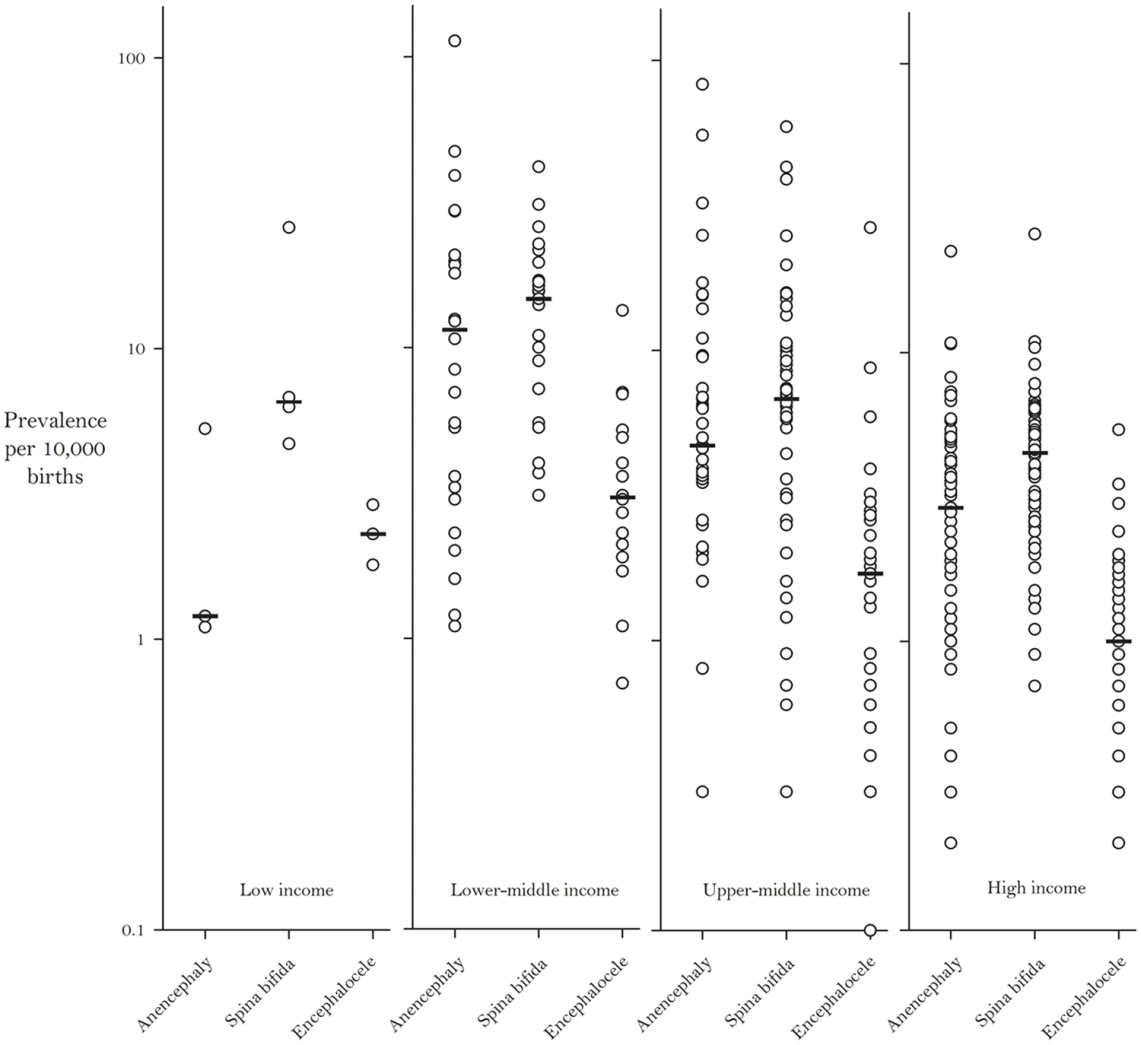
Prevalence per 10,000 Births for Specific Types of Neural Tube Defects by World Bank Income Classifications [18]. Bars Indicate the Median Prevalence for Each Condition.

**Fig 5:**
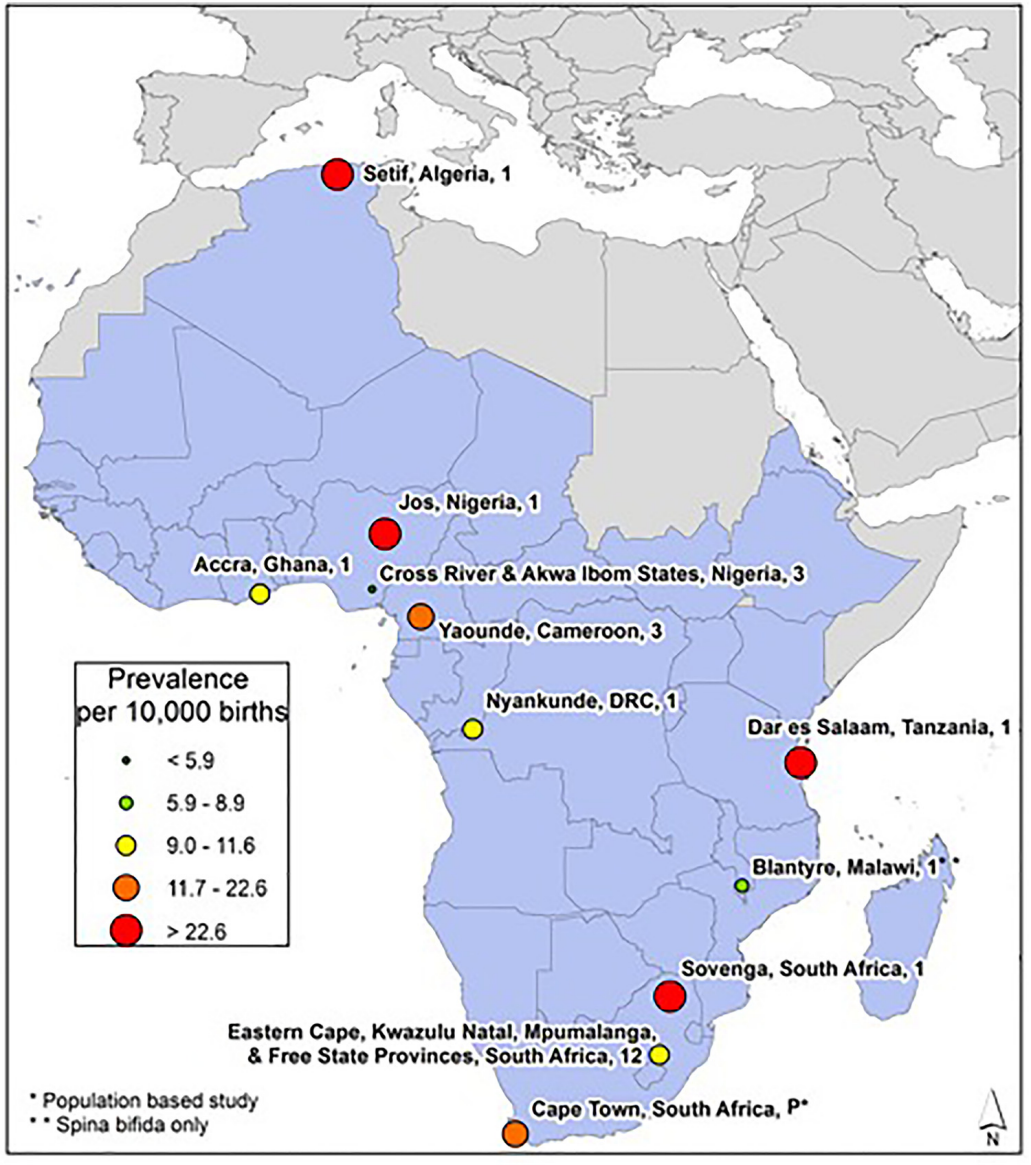
African Region Neural Tube Defects Prevalence Estimates (Location, Number of Hospitals). If there were national data available for more than one NTD, the entire country was filled-in based on the prevalence per 10,000 births. In instances where multiple prevalence estimates were available at the national level, the prevalence reported by the study/report with the least risk-of-bias was selected. Countries colored in grey are not a part of the World Health Organization region. Shapefile reprinted from http://www.diva-gis.org under a CC BY license, with permission from DIVA-GIS and Dr. Robert Hijmans.

Furthermore, we observed that among studies that reported stratified data for all three types of NTDs, on average, spina bifida attributed the highest percentage to total NTD prevalence, followed by anencephaly and then encephalocele (Fig 3). When stratified by country income level, we noticed a general decrease in the median prevalence for each specific type of NTD from the lower-middle to high income countries (Fig 4). NTD prevalence estimates by WHO region are as follows:

African Region: Data from eight of 47 WHO member countries, represented by 11 studies, met our inclusion criteria (Fig 5). The lowest reported NTD prevalence for the region was reported in Nigeria (5.2 per 10,000 births) [24] and the highest was reported in Algeria (75.4 per 10,000 births) [19]. The median NTD prevalence was 11.7 per 10,000 births. Data from this region were primarily gathered from hospital-based retrospective case reviews.

**Fig 6:**
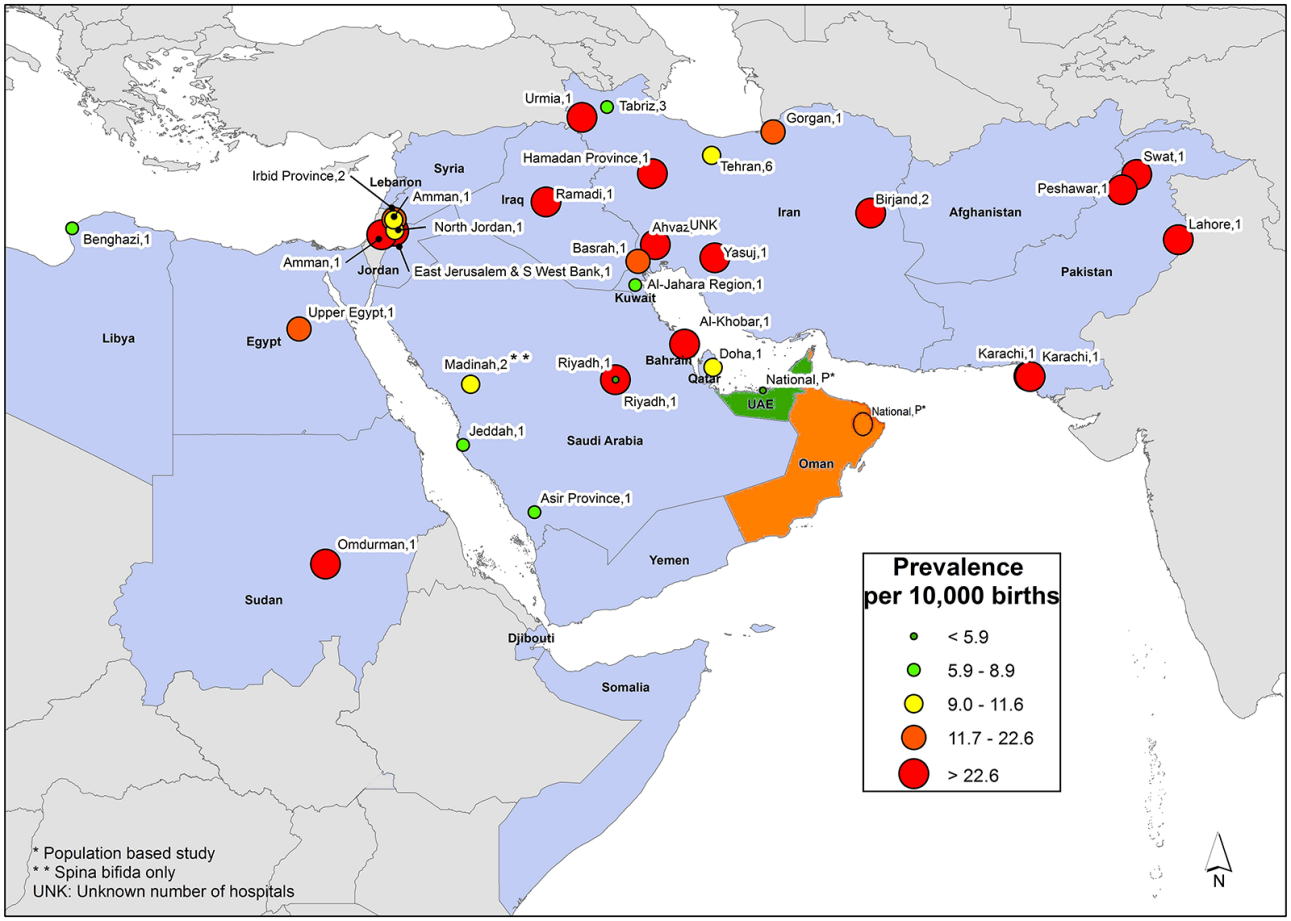
Eastern Mediterranean Region Neural Tube Defects Prevalence Estimates (Location, Number of Hospitals). If there were national data available for more than one NTD, the entire country was filled-in based on the prevalence per 10,000 births. In instances where multiple prevalence estimates were available at the national level, the prevalence reported by the study/report with the least risk-of-bias was selected. Countries colored in grey are not a part of the World Health Organization region. Shapefile reprinted from http://www.diva-gis.org under a CC BY license, with permission from DIVA-GIS and Dr. Robert Hijmans.

Eastern Mediterranean Region: Published data were available for 12 of the 21 countries in the region and were represented by 31 studies (Fig 6). This region exhibited variability in reported NTD prevalence as well, with estimates as low as 2.1 per 10,000 births in the United Arab Emirates [60] and as high as 124.1 per 10,000 births in Swat, Pakistan [48]. This region had the highest median prevalence (21.9 per 10,000 births). Elevated NTD prevalence estimates were consistently observed in Pakistan. All five studies in Pakistan reported estimates between 38.6 and 124.1 per 10,000 births [48–52].

**Fig 7:**
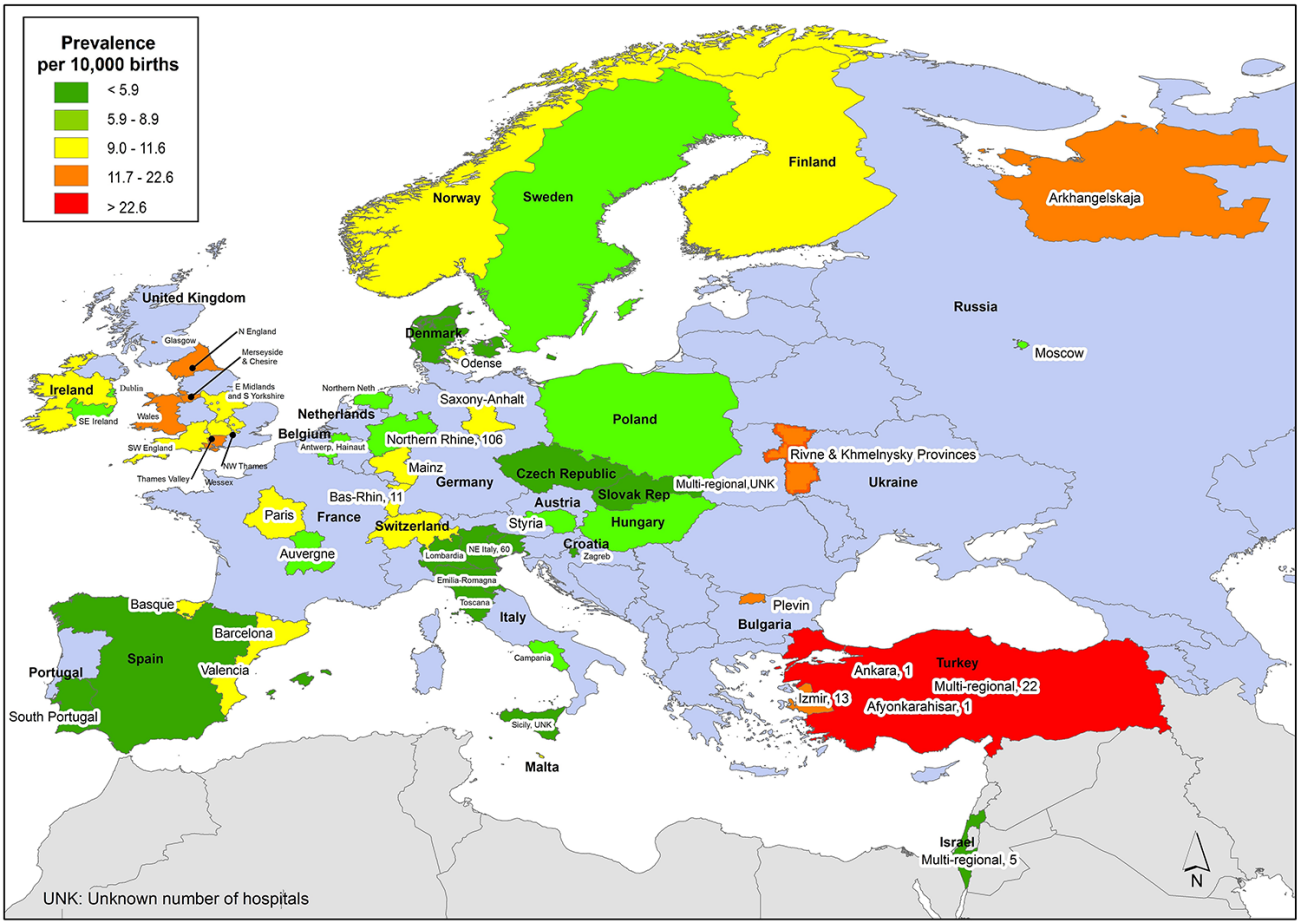
European Region Neural Tube Defects Prevalence Estimates (Location, Number of Hospitals). The majority of data from the European region was population based. All data based on hospital studies from regions is indicated with the number of hospitals. If there were national or regional data available for more than one NTD, the entire country or region was filled-in based on the prevalence per 10,000 births. In instances where multiple prevalence estimates were available at the national level, the prevalence reported by the study/report with the least risk-of-bias was selected. Countries colored in grey are not a part of the World Health Organization region. A national study from Israel is not represented on this map since it only provided prevalence by ethnicity. Shapefile reprinted from http://www.gadm.org under a CC BY license, with permission from Global Administrative Areas and Dr. Robert Hijmans.

European Region: We identified a total of 60 different studies/reports spanning a total of 26 countries of the 53 countries in the region (Fig 7). Ninety-five percent of NTD data from Europe came from regional or national registries/surveillance systems. The reported NTD prevalence estimates in this region were relatively less variable than other regions (range: 1.3–35.9 per 10,000 births) [61, 70]. The median for the European region was 9.0 per 10,000 births.

**Fig 8:**
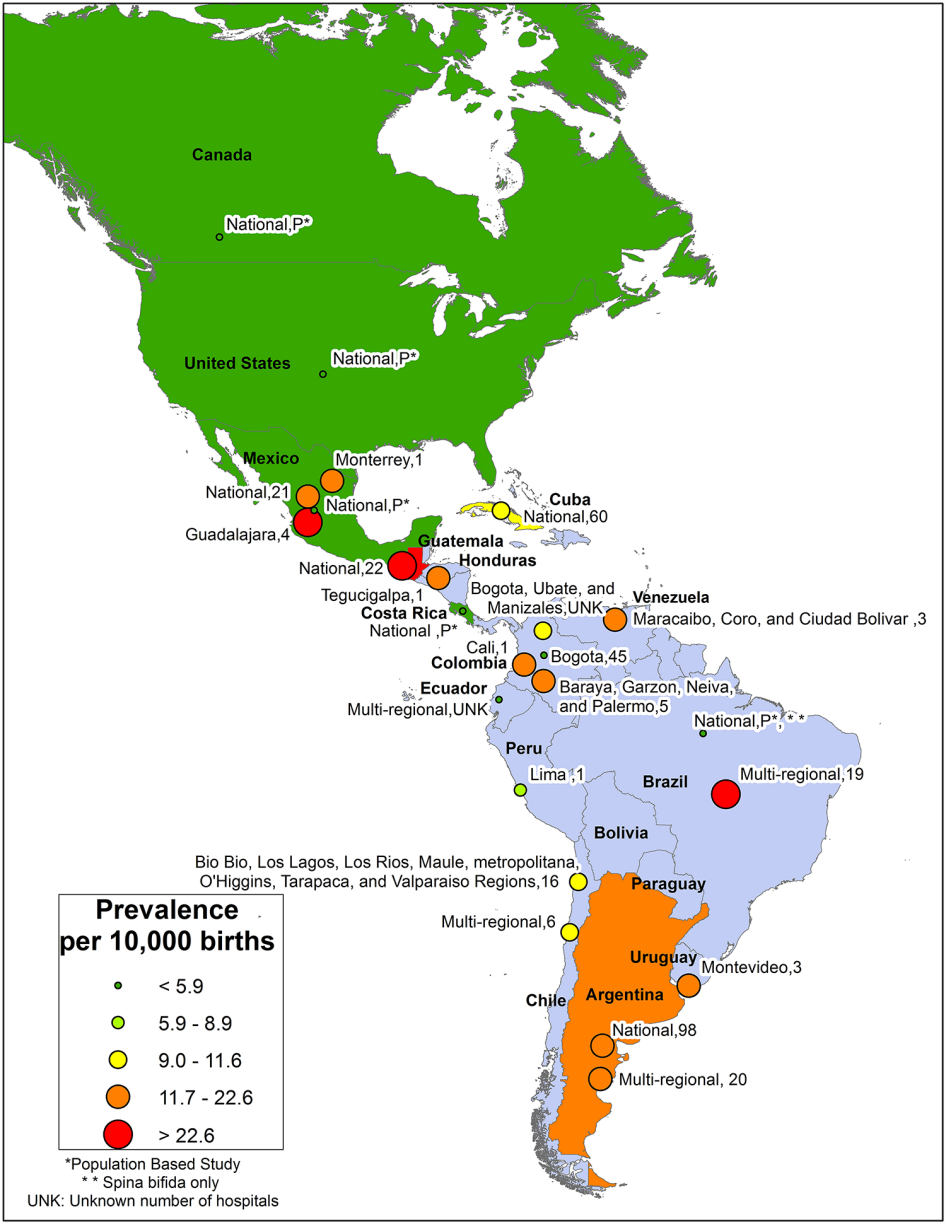
American Region Neural Tube Defects Prevalence Estimates (Location, Number of Hospitals). If there were national data available for more than one NTD, the entire country was filled-in based on the prevalence per 10,000 births. In instances where multiple prevalence estimates were available at the national level, the prevalence reported by the study/report with the least risk-of-bias was selected. Shapefile reprinted from http://www.diva-gis.org under a CC BY license, with permission from DIVA-GIS and Dr. Robert Hijmans.

Americas Region: Data from 21 studies/reports representing 15 of the 35 countries were available (Fig 8). This region had the least variability in reported NTD prevalence estimates. Among studies that included spina bifida and at least one other NTD, the lowest prevalence was 3.3 per 10,000 births [82, 87]. A study from Brazil which only counted spina bifida reported a prevalence of 1.4 per 10,000 births [75]. In this region, the highest prevalence was reported in Guatemala (27.9 per 10,000 births) [83]. The median prevalence was 11.5 per 10,000 births.

**Fig 9:**
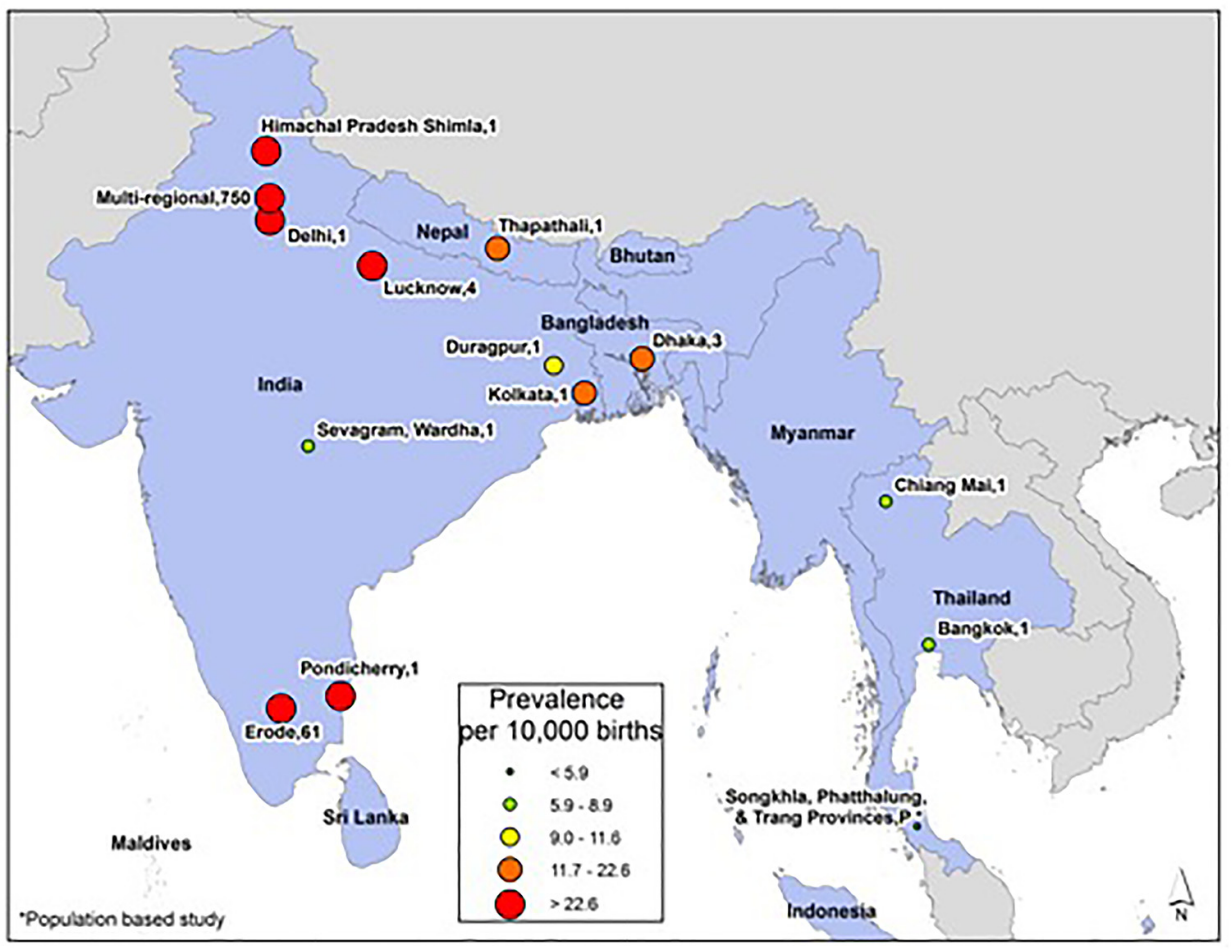
South-East Asian Region Neural Tube Defects Prevalence Estimates (Location, Number of Hospitals). If there were national data available for more than one NTD, the entire country was filled-in based on the prevalence per 10,000 births. In instances where multiple prevalence estimates were available at the national level, the prevalence reported by the study/report with the least risk-of-bias was selected. North Korea had no reported data and was not shown in map due to scaling considerations. Shapefile reprinted from http://www.diva-gis.org under a CC BY license, with permission from DIVA-GIS and Dr. Robert Hijmans.

South-East Asian Region: There were 14 studies representing four of the 11 countries in South-East Asia (Fig 9). The lowest prevalence estimate for the region was 1.9 per 10,000 births in Thailand [101] and the highest was 66.2 per 10,000 births in India [93]. Most of the data for this region came from either Thailand or India; three and nine studies, respectively. The median prevalence in this region was 15.8 per 10,000 births.

**Fig 10:**
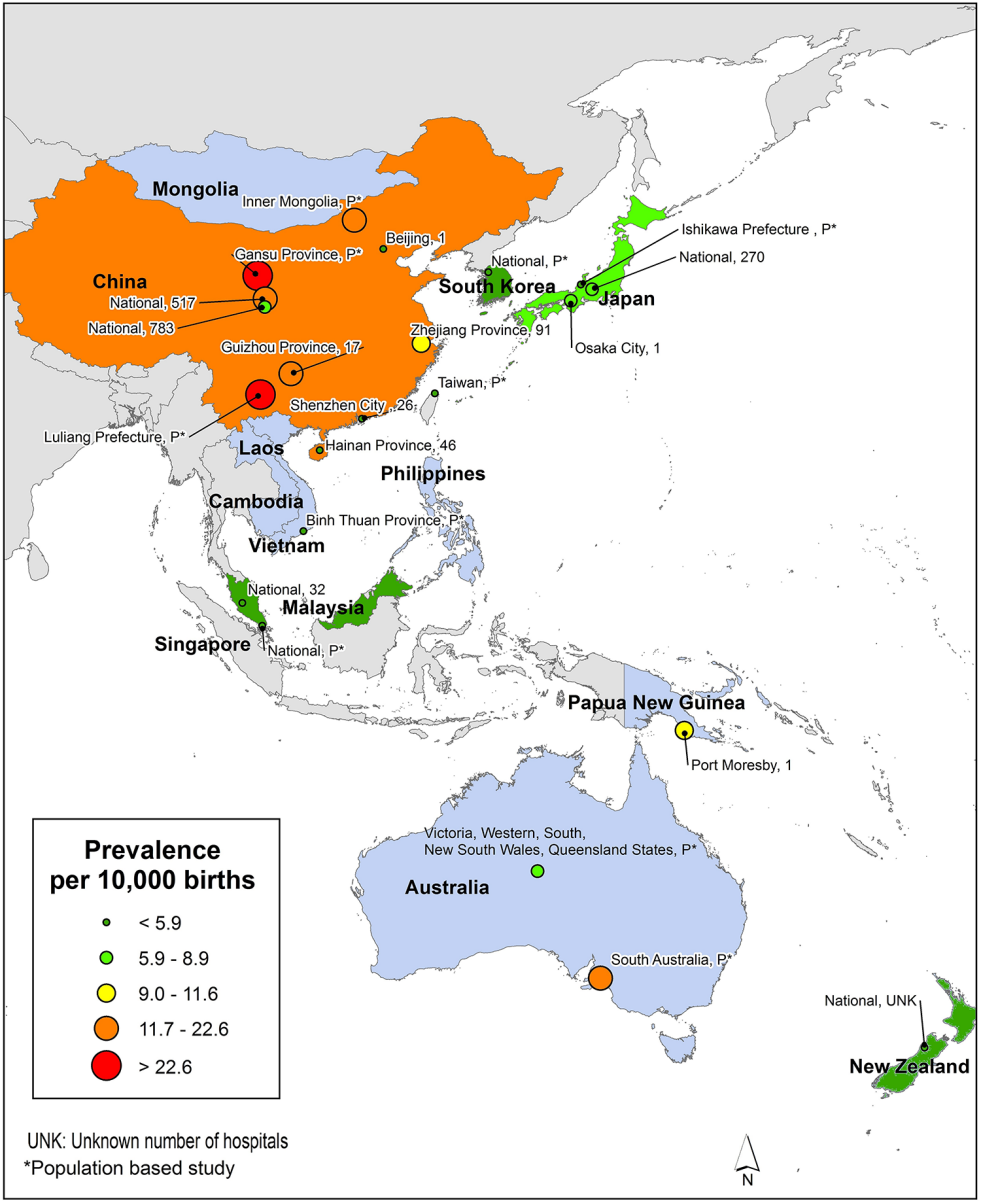
Western Pacific Region Neural Tube Defects Prevalence Estimates (Location, Number of Hospitals). If there were national data available for more than one NTD, the entire country was filled-in based on the prevalence per 10,000 births. In instances where multiple prevalence estimates were available at the national level, the prevalence reported by the study/report with the least risk-of-bias was selected. Countries colored in grey are not a part of the World Health Organization region. Shapefile reprinted from http://www.diva-gis.org under a CC BY license, with permission from DIVA-GIS and Dr. Robert Hijmans.

Western Pacific Region: Of the 27 countries, data were available for nine countries from 22 studies/reports (Fig 10). This region had the lowest median prevalence value (6.9 per 10,000 births). As stated previously, China exhibited the greatest variability in reported NTD prevalence estimates (range: 0.3–199.4 per 10,000 births) [113, 112]. As seen in Li et al., NTD estimates tend to be higher in northern China (18.7 per 10,000 births) than in the southern part of the country (9.7 per 10,000 births) [108]. When excluding data from China, reported NTD prevalence in this region ranged from as low as 1.2 per 10,000 births in Singapore [122] to as high as 19.5 per 10,000 births in Australia [104].

### Surveillance System/Registry Coverage

**Fig 11:**
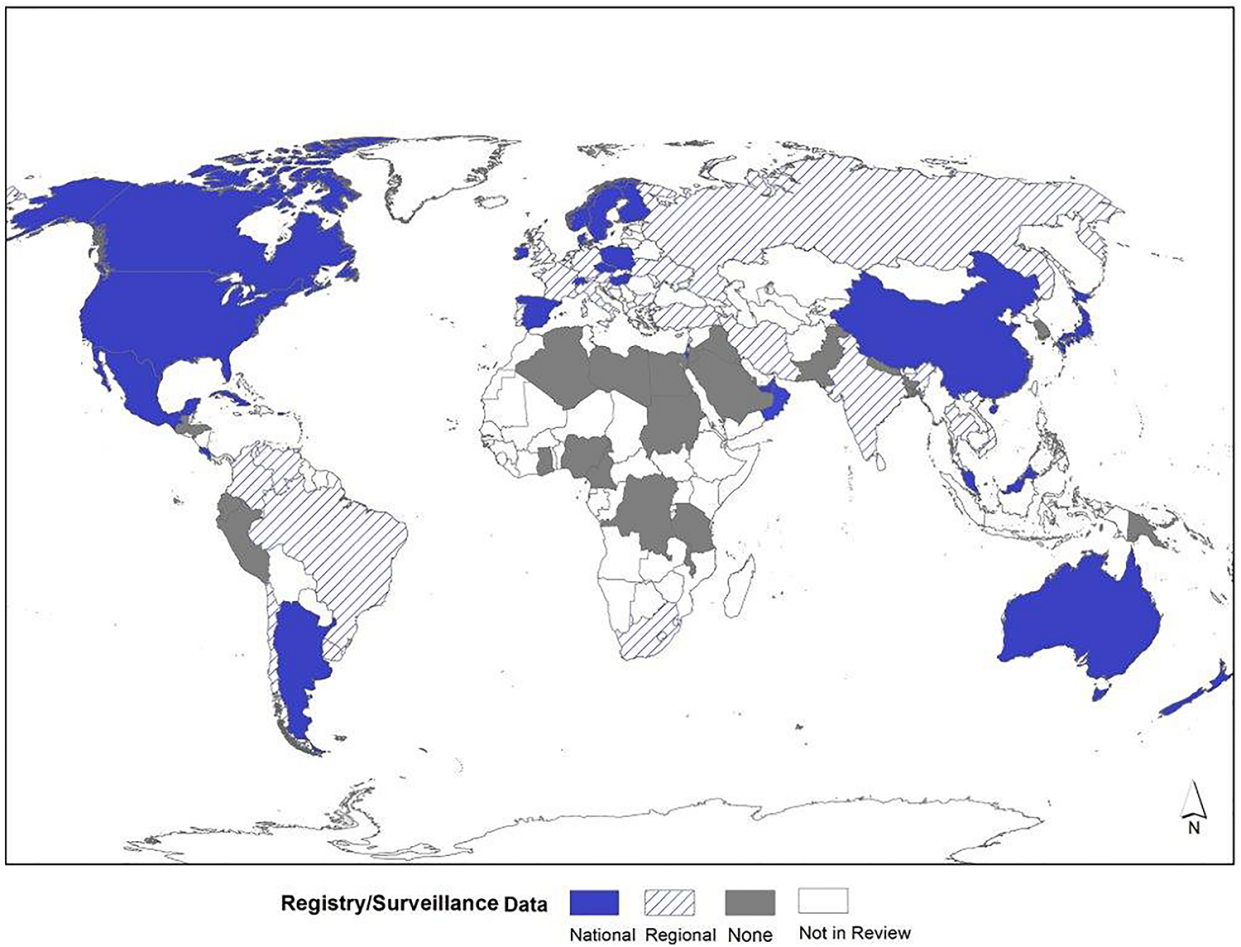
Data Source: Surveillance/Registry Coverage by Geographic Level. Shapefile reprinted from http://www.diva-gis.org under a CC BY license, with permission from DIVA-GIS and Dr. Robert Hijmans.

Fig 11 shows the types of NTD data collection worldwide, categorized as national surveillance system/registry, regional surveillance system/registry, or other (i.e., no surveillance system/registry but has data collected using another methodology). The map illustrates that there are limited amounts of data derived from surveillance/registry programs in countries in the African (1/8) and South-East Asian (2/4) regions. In contrast, the Americas (11/15) and European (26/26) countries had higher utilization of surveillance/registries. Furthermore, the presence of a NTD surveillance system/registry increased with country income status: low income (0%), lower-middle (25%), upper-middle (70%), and high income (91%).

### Risk-of-Bias (RoB)

**Fig 12:**
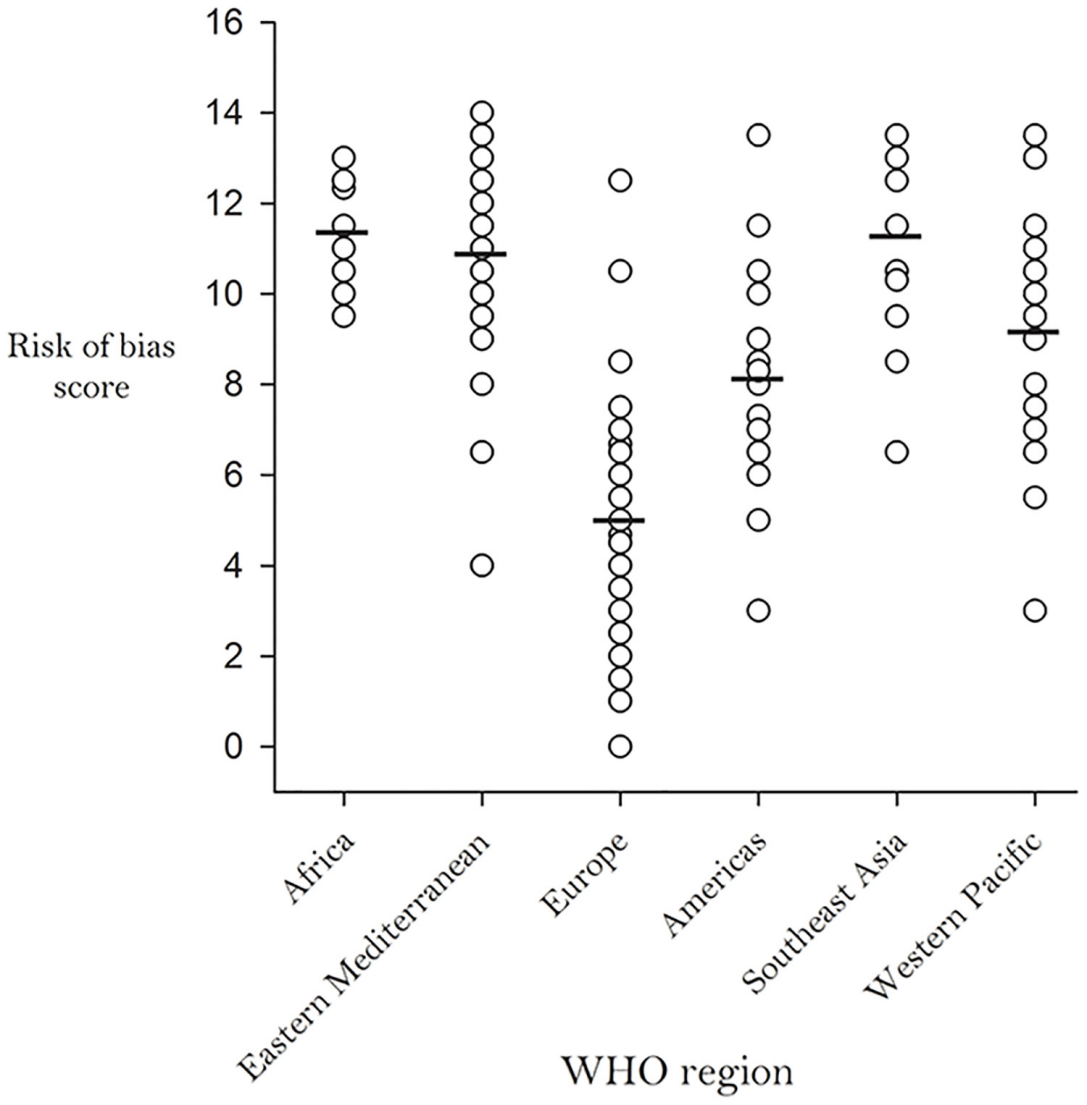
Average Study Risk-of-Bias by World Health Organization Region.

**Fig 13:**
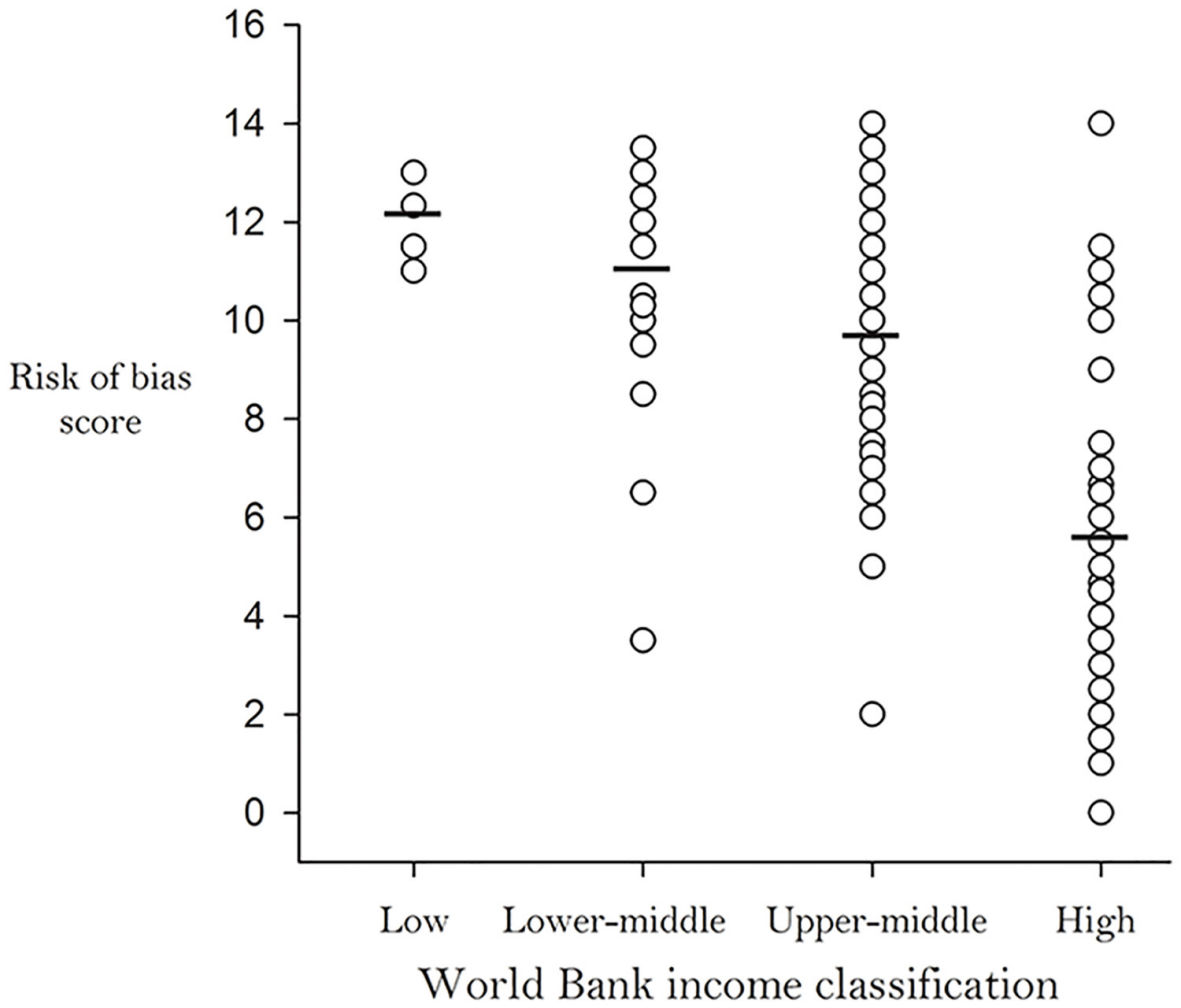
Average Study Risk-of-Bias by World Bank Income Classification [18].

The RoB evaluation generated scores ranging from 0.0 to 14.0 (possible range 0.0 to 18.0), with lower scores indicating lower RoB. When average RoB scores were classified by WHO region, studies/reports from Europe had the lowest (5.0), while studies/reports from the Eastern Mediterranean (10.9), South-East Asian (11.3) and African (11.5) regions had the highest RoB scores (Fig 12). In addition, we observed an inverse relationship between RoB score and country income level. As the income level of countries increased, their average RoB scores decreased (Fig 13).

## Discussion

Our review provides a comprehensive global assessment of NTD prevalence as observed from 75 countries at the national, regional, or local levels, which represents about 40% of the total number of WHO member states (194) [125]. The African and South-East Asian regions have minimal data available, demonstrating the need to establish surveillance and other mechanisms that can provide countries with standardized data to better determine the burden of birth defects in general, and NTDs in particular. More complete ascertainment of data will be useful in determining country level needs for prevention of NTDs, monitoring trends through time, helping to evaluate the impact of prevention efforts, and developing services for those affected.

Overall, reported prevalence estimates varied greatly between, and also, within countries ranging from 0.3 to 199.4 NTDs per 10,000 births. Through the RoB assessment, we discovered this may be in part due to variation in data collection methodology among individual studies. For example, both studies from post-fortification Brazil had a 10-fold difference in spina bifida prevalence estimates: 1.4 per 10,000 live births (95% CI: 1.2, 1.5) in the Orioli et al. study [76] and 14.2 per 10,000 births (95% CI: 11.8, 16.6) in the Lopez-Camelo et al. study [75]. Orioli et al. assessed spina bifida prevalence in 2006 in a population-based cross-sectional study that included millions of live births from the Live Births Information System. The system used to estimate NTDs in the Orioli et al. paper had some limitations with case ascertainment, case definition, and lack of standardized diagnoses that may impact the validity and reliability of the estimates [76, 126]. The Lopez-Camelo et al. study used data from the Latin American Collaborative Study of Congenital Anomalies (ECLAMC) which is a hospital-based, voluntary birth defects surveillance network that includes 19 hospitals throughout Brazil. It is important to note that the NTD prevalence variability we found in our review could also be true differences, resulting from other factors including nutritional factors, genetics, routine folic acid supplementation, and the presence of folic acid fortification programs [127–129].

By conducting our RoB assessment, we found that case ascertainment methods and data quality varied greatly among studies. Therefore, the prevalence estimates from different studies are not directly comparable nor can they be used to calculate a combined estimate [130]. For example, the scope of studies varied from single-hospital studies done over the span of one year to studies using established nationally representative surveillance systems. In addition, many studies did not clearly define NTDs or provide inclusion criteria (e.g., gestational age and birth outcome). While we attempted to re-calculate reported prevalence to match our definition (e.g., removing chromosomal NTDs and spina bifida occulta), many times this was not possible because data were not stratified by type of NTD. Standardized protocols (i.e., case definitions, inclusion criteria, variables collected, reporting) for birth defects surveillance would allow better comparison among studies. In addition, improved methodology can make prevalence estimates more accurate. For example, including cases among pregnancies terminated for fetal anomalies, especially in countries where this is legal, usually leads to higher and more accurate prevalence estimates due to better case ascertainment. Recently, standardized tools for birth defects surveillance have been developed through a collaborative effort of health organizations including WHO, CDC, and ICBDSR. The Birth Defects Surveillance Manual and Atlas of Selected Congenital Anomalies are available in three languages (English, Spanish, and French) and have been developed specifically for low and middle income countries [131, 132].

In our review, although some data were available from low and middle income countries, most of the data were not derived from surveillance systems or registries. Often data from these countries were collected in limited geographic areas (single hospital studies), were not population-based, and lacked well defined procedures for collecting birth defects data. NTD prevalence data from surveillance systems and registries, such as EUROCAT, that used standardized and more comprehensive case ascertainment protocols (e.g., reporting cases from termination of pregnancy where it is legal) and had greater geographic and population coverage are more likely to estimate the true burden of NTDs in those regions more accurately.

This review advances the state of knowledge in three ways: first, this is the most current systematic review on global NTD prevalence; second, this review was able to identify large gaps in data collection and highlight international differences; and third, through the RoB assessment this study was able to document the wide variation in the quality and methodology of current reports. Our review supports the findings of previously published literature and demonstrates there is a high burden of NTDs globally. However, our review purposefully does not model data to non-reporting regions in an effort to highlight the lack of data globally. Moreover, it expands the scope of previously published systematic reviews that only included studies/reports from countries in one region or select income levels.

## Limitations

Beyond issues related to the abstracted data and study-specific methodologic issues, our review is also limited by factors related to our search criteria. Since this review only searched English and Spanish literature and excluded studies with small study populations, it may not have incorporated all relevant NTD prevalence information. In select studies, our review was unable to report prevalence estimates for each specific type of NTD since individual values were not always stratified. Lastly, presence of birth outcome data (i.e., live birth, stillbirth, and termination of pregnancy) was only used for the RoB analysis. Ideally, prevalence data should be stratified by birth outcome, however, many studies did not describe the birth outcome in sufficient detail (i.e., whether it was in the numerator, denominator, or both) or at all.

## Conclusions

This review describes the available data on the current burden of NTDs throughout the world. Despite methodological variations and coverage gaps in data collection, high NTD prevalence estimates throughout the literature indicate that NTDs remain an important preventable public health problem. This review provides a snapshot of areas in need of greater coverage and quality of NTD monitoring and surveillance and identifies opportunities for development such as standard reporting of birth defects as recommended by the World Health Assembly resolution. More importantly, regions that include large portions of the global population (e.g., South-East Asia) are lacking surveillance/registry data and case ascertainment methods that include all birth outcomes which provide the most reliable and valid estimates. In response to this need, CDC’s Birth Defects COUNT global initiative is working with partners in South-East Asia, East and Central Africa, and Latin America to implement and improve surveillance of NTDs as well as other birth defects [133].

## Supporting Information

S1 Document PRISMA Checklist. (DOC)

S2 Document Permission to publish map shapefiles. (DOCX)
