# Supplementary material for: Describing the Prevalence of Neural Tube Defects Worldwide: A Systematic Literature Review
Source: PLoS One. 2016 Apr 11;11(4):e0151586. doi: 10.1371/journal.pone.0151586 (PMC4827875; doi:10.1371/journal.pone.0151586)
Supplement: S2 Document — (DOCX) [file pone.0151586.s002.docx]

S2 Document. Permission to publish map shapefiles

**From:** Robert Hijmans [<mailto:rhijmans@ucdavis.edu>]
**Sent:** Monday, February 01, 2016 11:58 AM
**To:** Zaganjor, Ibrahim (CDC/ONDIEH/NCBDDD) (CTR) <[wwa3@cdc.gov](mailto:wwa3@cdc.gov)>
**Subject:** Re: Permission to publish maps/how to cite

Dear Ibrahim,

You have permission to use these data to create maps and publish them in PLOS ONE under the Creative Commons Attribution License (CCAL) CC BY 4.0 (<http://creativecommons.org/licenses/by/4.0/>).

As for citation, it is nice if you provide a link to the source, but that is up to you.

Robert

--

Robert J. Hijmans
Associate Professor, Department of Environmental Science and Policy

Chair, Geography Graduate Group

Director, Geospatial and Farming Systems Consortium, Feed the Future Sustainable Intensification Innovation Lab

University of California, Davis

2001 Wickson Hall
(530) 752-6555

On Mon, Feb 1, 2016 at 8:52 AM, Zaganjor, Ibrahim (CDC/ONDIEH/NCBDDD) (CTR) <[wwa3@cdc.gov](mailto:wwa3@cdc.gov)> wrote:

Dear Dr. Hijmans,

I hope this email finds you well. My name is Ibrahim Zaganjor, a health scientist with the Centers for Disease Control and Prevention. I recently submitted a manuscript for publication to PLOS ONE using shapefiles available on two of your websites, Global Administrative Areas (<http://gadm.org/country>) and DIVA-GIS (<http://www.diva-gis.org/Data>). As a part of our revise and resubmit, PLOS ONE has requested we seek permission to publish these materials under the specific Creative Commons Attribution License (CCAL) CC BY 4.0.

I request permission for the open-access journal PLOS ONE to publish maps under the Creative Commons Attribution License (CCAL) CC BY 4.0 (<http://creativecommons.org/licenses/by/4.0/>). Please be aware that this license allows unrestricted use and distribution, even commercially, by third parties. Please reply and provide explicit written permission to publish these maps under a CC BY license.

Also, if this falls under academic use, how would you like us to cite/credit you as a source for the maps?

Best,

Ibrahim Zaganjor
